# Hyperinvasiveness of *Salmonella enterica* serovar Choleraesuis linked to hyperexpression of type III secretion systems *in vitro*

**DOI:** 10.1038/srep37642

**Published:** 2016-11-25

**Authors:** Kuan-Yeh Huang, Yi-Hsin Wang, Kun-Yi Chien, Rajendra Prasad Janapatla, Cheng-Hsun Chiu

**Affiliations:** 1 Graduate Institute of Biomedical Sciences, Chang Gung University College of Medicine, Taoyuan, Taiwan; 2 Molecular Infectious Disease Research Center, Chang Gung Memorial Hospital, Chang Gung University College of Medicine, Taoyuan, Taiwan

## Abstract

*Salmonella enterica* serovars Choleraesuis and Typhimurium are among the non-typhoid *Salmonella* serovars that are important zoonotic pathogens. In clinical observation, *S.* Typhimurium typically causes diarrheal diseases; however, *S.* Choleraesuis shows high predilection to cause bacteremia. The mechanism why *S.* Choleraesuis is more invasive to humans remains unknown. In this study, we compared the *S.* Typhimurium LT2 and *S.* Choleraesuis SC-B67 proteomes through stable isotope labeling of amino acid in cell culture (SILAC). In SILAC, the expression of many virulence proteins in two type III secretion systems (T3SSs) were significantly higher in *S.* Choleraesuis than in *S.* Typhimurium. Similar differences were also found at the transcriptional level. Compared to *S.* Typhimurium, *S.* Choleraesuis showed a higher penetration level to Caco-2 (>100-fold) and MDCK (>10-fold) monolayers. In mice after oral challenge, the invasion of spleen and liver was also higher in *S.* Choleraesuis than in *S.* Typhimurium. The transcription of *hilD* in *S.* Choleraesuis was increased in physiological (1 mM) or high (10 mM) concentrations of Mg^2+^, but not in low (8 μM) concentration. We conclude that *S.* Choleraesuis showed hyperinvasiveness in cellular as well as mouse models due to hyperexpression of T3SS genes.

*Salmonella* is one of the most important pathogens to humans. *Salmonella* can be divided into typhoid and non-typhoid *Salmonella* (NTS) serovars according to the respective clinical syndromes. Both *S.* Typhimurium and *S.* Choleraesuis are NTS serovars. *S.* Typhimruium typically causes diarrheal disease in young children, while *S.* Choleraesuis frequently causes diseases in all ages^1^,^2^. Previous studies have demonstrated the high invasion ability of *S.* Choleraesuis through various *ex vivo* and *in vitro* assays^3^,^4^. However, the mechanism underlying such high pathogenicity in *S.* Choleraesuis remains unknown.

The most important virulence mechanism in the *Salmonella* is the type III secretion system (T3SS). *Salmonella* contains two sets of T3SS, the genes of which are located in *Salmonella* pathogenicity island-1 and -2 (SPI-1 and SPI-2)^5^,^6^,^7^. SPI-1 T3SS participates in the early stage of infection in *Salmonella* adhesion and invasion^8^, while SPI-2 T3SS is involved in replication of *Salmonella* in both the phagocytic and non-phagocytic cells^9^. SPI-2 T3SS also plays an important role in the maturation of *Salmonella*-containing vacuoles (SCVs)^10^. The regulation of SPI-1 and SPI-2 T3SS is controlled by different mechanisms, in which HilA and HilD are two important proteins that can activate SPI-1 T3SS^11^,^12^,^13^,^14^. Environmental signals, like Mg^2+^, also regulate the SPI-1 and SPI-2 T3SS expression through two-component signal transduction systems (TCSs)^15^,^16^.

To elucidate why *S.* Choleraesuis is more invasive than *S.* Typhimurium, we compared protein expression pattern between the two serovars through stable isotope labeling by amino acids in cell culture (SILAC) analysis. We found that virulence factors linked to invasiveness were highly expressed in *S.* Choleraesuis. *S.* Choleraesuis appeared more pathogenic in cellular and murine models. Under normal and high concentrations of Mg^2+^, the expression of SPI-1 was even higher in *S.* Choleraesuis than in *S.* Typhimurium. It appears that the presence of Mg^2+^ is associated with the higher expression of SPI-1 virulence factors in *S.* Choleraesuis, thereby promoting the subsequent invasion and penetration of *S.* Choleraesuis through host cells.

## Results

### Comparison of the protein expression patterns between *S.* Choleraesuis and *S.* Typhimurium

In the differential proteomic study, 1731 proteins were identified in both *S.* Typhimurium LT2 and *S.* Choleraesuis SC-B67. 287 (16.6%) proteins were expressed two times higher in *S.* Choleraesuis SC-B67 than in *S.* Typhimurium LT2; on the other hand, 183 (10.6%) were expressed two times higher in *S.* Typhimurium LT2 than in *S.* Choleraesuis SC-B67 in RPMI cell culture medium (Supplementary Tables S3 and S4). Expression level of major virulence factors involved in *Salmonella* pathogenesis was higher in *S.* Choleraesuis SC-B67 than in *S.* Typhimurium LT2 (35/287, 12.20%; Table 1). The 35 virulence genes were mainly located in the virulence plasmid, SPI-1, SPI-2, SPI-4, SPI-5, and SPI-11 (Table 1). The transcriptional level of *hilA* and *hilD* was 24.0 and 21.1 fold, respectively, higher in *S.* Choleraesuis than in *S.* Typhimurium. Similar results were obtained in both the media RPMI 1640 and DMEM (Supplementary Figure S1). The transcription level of *sipB*, an effector protein gene for SPI-1 T3SS, was also higher in *S.* Choleraesuis, this correlates to the protein expression level (Supplementary Figure 1). In addition to strains used in SILAC, clinical isolates of *S.* Choleraesuis also expressed higher level of *hilD* (Supplementary Figure 2).

### Acid tolerance

At 10 minutes post inoculation in simulated gastric fluid (SGF, pH 2.0), the number of alive bacteria was 100-fold higher in *S.* Choleraesuis than in *S*. Typhimurium (Supplementary Figure 3). At 10–30 minutes of incubation, *S.* Choleraesuis SC-B67 still outnumbered *S*. Typhimurium SL1344 by approximately 10-fold, although there was no significant difference after 15 minutes. (Supplementary Figure 3), suggesting that *S.* Choleraesuis showed better acid tolerance than *S.* Typhimurium.

### HeLa (non-polar) and MDCK (polar) cell invasion

To compare the invasion ability between *S.* Typhimurium and *S.* Choleraesuis, we infected the epithelial cells with the two serovars separately. *S.* Choleraesuis and *S.* Typhimurium showed no difference in the level of invasiveness to polar and non-polar cells (Supplementary Figure 4).

### Penetration to epithelial cells

**Figure 1:**
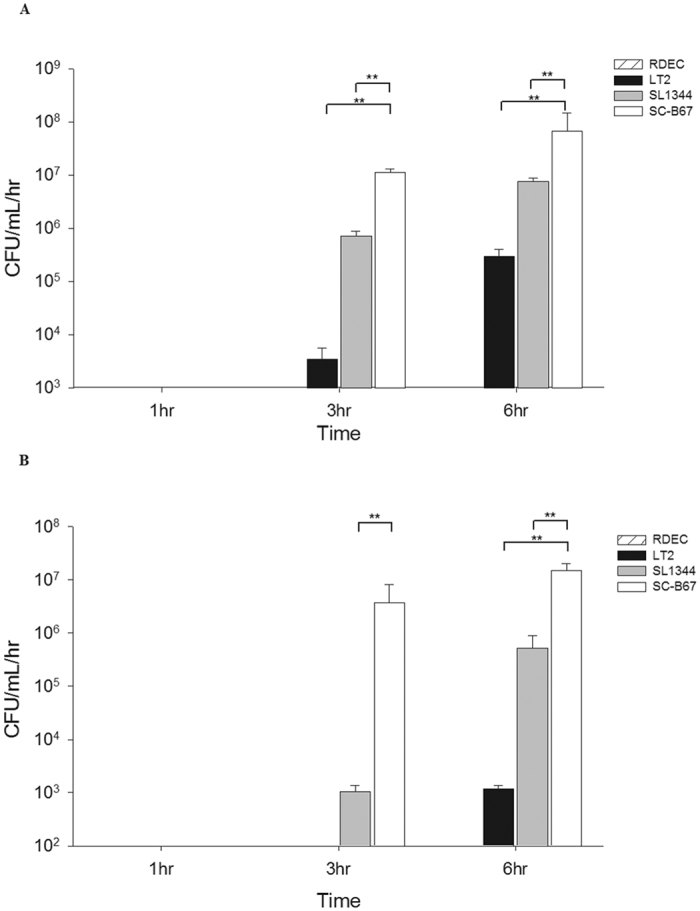
Penetration assay of *S.* Typhimurium SL1344 and *S.* Choleraesuis SC-B67 through cellular monolayer. The cells used were MDCK (**A**) and Caco-2 (**B**) cells. At appropriate time points, the number of *Salmonella* was calculated by plating on LB agar. The experiment was repeated 3 times. **p < 0.01.

*S.* Choleraesuis penetrated the MDCK (10-fold) and Caco-2 cell (1000-fold) monolayers at higher efficiency than *S.* Typhimurium (Fig. 1) at 3 hours post-infection. Even at 6 hours after infection, the number of bacteria penetrating the monolayer was still higher in *S.* Choleraesuis than in *S.* Typhimurium, especially in Caco-2 cells (Fig. 1). *S.* Choleraesuis clinical isolates also showed higher penetration ability than *S.* Typhimurium clinical isolates (Supplementary Figure 5).

### Intra-macrophage survival

**Figure 2:**
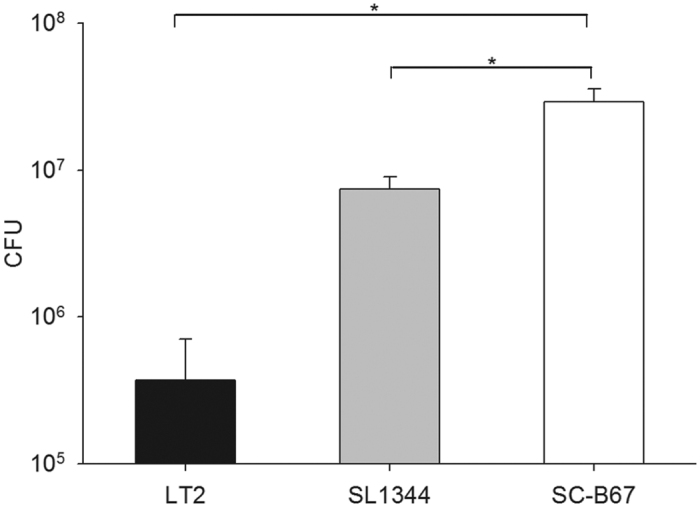
Intra-macrophage survival assay. THP-1 cells were infected by *S.* Choleraesuis SC-B67 and *S.* Typhimurium LT2 and SL1344. After 1 hour post-infection, cells were treated with gentamicin for 30 minutes. After 4 hours post-infection, cells were lysed and plated on LB agar. The experiment was repeated 3 times. *p < 0.05.

We compared the intra-macrophage survival between *S.* Typhimurium and *S.* Choleraesuis by using THP-1 cells. The result showed that *S.* Choleraesuis displayed at least 4-times higher survival than *S.* Typhimurium inside macrophages (Fig. 2). *S.* Choleraesuis clinical isolates also showed better intra-macrophage survival ability at 4 hours and 6 hours post-infection than *S.* Typhimurium clinical isolates (Supplementary Figure 6).

### Mouse experiments

**Figure 3:**
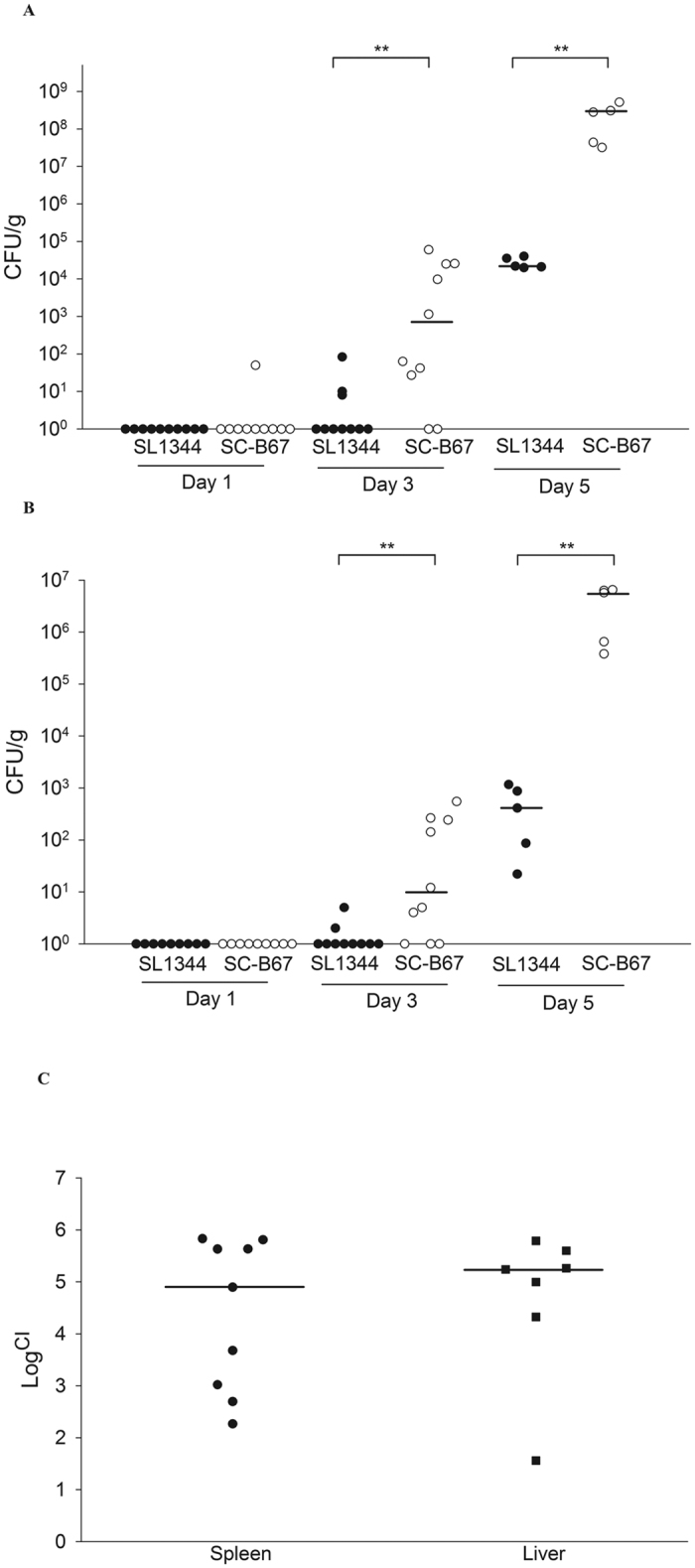
Isolation of *Salmonella* from the organs. BALB/c mice were infected by different *Salmonella* serovars through oral infection. At 1, 3, 5, and 6 day(s) post-infection, mice were sacrificed. Spleen (**A**) and liver (**B**) were grounded and plated on the SS agar plates. Mice were co-infected with SL1344 and SC-B67 (**C**). At 5 days post infection, both serovars were recovered from the spleen and liver, and analyzed by CI as described in the text. **p < 0.01.

*S.* Choleraesuis showed higher invasion and intracellular survival compared to *S.* Typhimurium in cellular experiments. We then compared the pathogenesis of the two serovars using a murine model. One day post infection, *S.* Choleraesuis was found in spleen of one mouse (1/10) (Fig. 3A). No bacteria (less than 10 CFU/g) was found in spleen and liver of other mice (Fig. 3A and B). At 3 days post-infection, bacteria was recovered from spleen and liver at least 10 CFU/g in seven mice infected by *S.* Choleraesuis, but only in three mice infected by *S.* Typhimurium (Fig. 3A and B). Both serovars were recovered from the spleen and liver at 5 days post-infection. However, the bacterial numbers recovered were approximately 1000-times higher in *S.* Choleraesuis than in *S.* Typhimurium at 5 days post-infection (Fig. 3A and B). To further confirm the difference, we co-infected the mice with *S.* Typhimurium and *S.* Choleraesuis. After 5 days, *S.* Typhimurium was significantly outnumbered by *S.* Choleraesuis in liver and spleen (Fig. 3C).

### Expression of *hilA* and *hilD* in different *hilE* complemented strains

**Figure 4:**
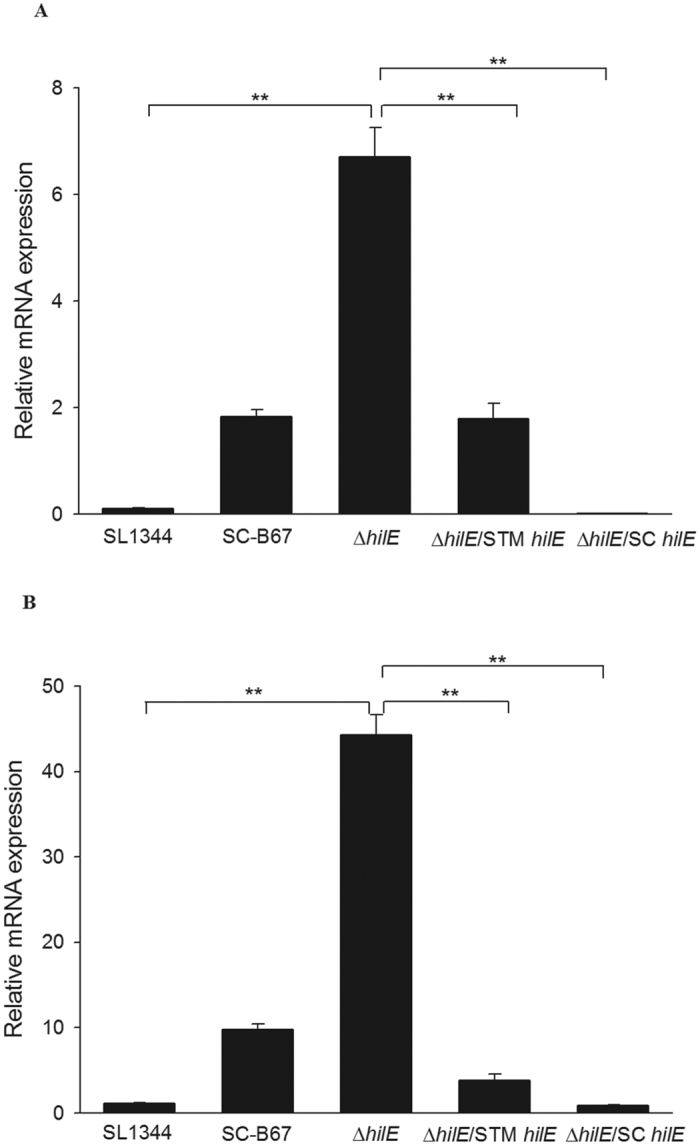
Expression of *hilA* and *hilD* in different *Salmonella* strains. All the bacterial strains were cultured in the DMEM for 6 hours. Total RNA were isolated and analyzed by qPCR. The expression level of *hilA* (**A**) and *hilD* (**B**) are shown relative to the 16S rDNA. **p < 0.01.

**Figure 5:**
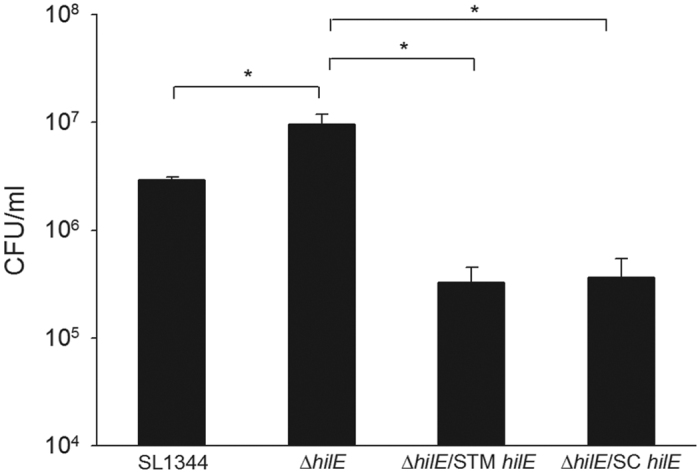
Intra-macrophage survival of *S.* Typhimurium SL1344 and its derivative strains. THP-1 cells were infected by SL1344, HilE mutant strains, and its complemented strains. Bacteria were recovered at 4 hours post-infection. *p < 0.05.

HilE is a negative regulator protein for SPI-1 T3SS virulence factors^17^. The predicted translation start site of *hilE* in *S.* Choleraesuis and *S.* Typhimurium is different due to the presence of an additional nucleotide between the two start codons in *S.* Choleraesuis (Supplementary Figure 7). HilE becomes a pseudogene in *S.* Choleraesuis if it uses the first start codon. The same difference was also found in the clinical *S.* Choleraesuis isolates (data not shown).We tried to construct the *hilE* deficient strain in both *S.* Typhimurium and *S.* Choleraesuis. However, all the *S.* Choleraesuis are multiple drug resistant strains, we do not have suitable selection markers to construct mutant strains. To analyze the function of HilE in *S.* Choleraesuis, we transformed a plasmid harboring *hilE* sequence from *S.* Choleraesuis into *hilE*-deficient strain in SL1344. The expression of *hilA* and *hilD* was very low in the deletion mutant carrying the *hilE* gene from either *S.* Typhimurium or *S.* Choleraesuis (Fig. 4). In the intra-macrophage survival assay, both *hilE* complemented strains showed poor survival (Fig. 5). The results indicated that the *hilE* gene from either *S.* Typhimurium or *S.* Choleraesuis was functional and thus can complement the *hilE* deletion to suppress the expression of the SPI-1 T3SS.

### Hypersensitivity to Mg^2+^ in *S.* Choleraesuis

**Figure 6:**
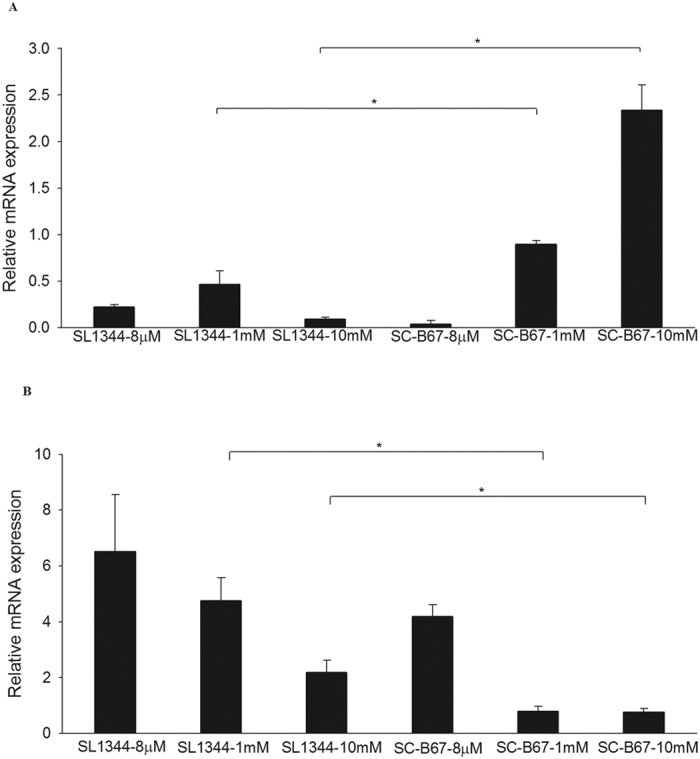
Gene expression of SL1344 and SC-B67 under different Mg^2+^ concentrations. Both serovars were cultured in N-Salt medium with 8 μM, 1 mM, and 10 mM Mg^2+^ concentrations. Then the total RNA were isolated and analyzed by qPCR. The expression level of *hilD* (**A**), and *pagC* (**B**) in both serovars was examined in different Mg^2+^ concentrations. All the expression levels are relative to the 16S rDNA. *p < 0.05.

Environmental signals, like oxygen, Mg^2+^ and Fe^3+^, also control the SPI-1 T3SS expression^11^,^15^,^18^. We checked whether or not the environmental signals were the cause for differential gene expression between *S.* Typhimurium and *S.* Choleraesuis. Under both high and low oxygen conditions, the level of *hilD* were higher expressed in *S.* Choleraesuis SC-B67, relative to *S.* Typhimurium (Supplementary Figure 8). Mg^2+^ is a ligand bound to PhoPQ TCS. Under low Mg^2+^ condition, PhoPQ TCS is activated to suppress *hilA* and *hilD* expression; it is contrary under high Mg^2+^ condition^15^. In low (8 μM) Mg^2+^ condition, the expression of *hilD* was low in both serovars (Fig. 6A). Interestingly, *hilD* expression was increased in *S.* Choleraesuis SC-B67 under normal (1 mM) and high (10 mM) Mg^2+^ condition (Fig. 6A). PhoPQ TCS in *S.* Choleraesuis SC-B67 appeared hypersensitive to environmental Mg^2+^, leading to higher expression of HilD. To support this, we tested expression patterns of the PhoP-activated genes (Pags) under different Mg^2+^ concentrations in the two serovars. Under low Mg^2+^ condition, the expression of *pagC* was similar between both serovars, but it was 5.4-fold lower in the *S.* Choleraesuis than in *S.* Typhimurium under normal Mg^2+^ condition, suggesting *S.* Choleraesuis was hypersensitive to Mg^2+^ to cause HilD activation (Fig. 6B).

## Discussion

Systemic approaches, like genomic, transcriptomic, and proteomic methods, have been used to compare the difference among different serovars of *S. enterica*^19^,^20^,^21^. In genomic studies, *S.* Typhimurium and *S.* Choleraesuis all contained well-defined virulence genes in SPI-1 and SPI-2^19^. It is hard to use sequence comparison to explain why *S.* Choleraesuis caused more bacteremia than other serovars in humans. Previous studies compared the protein expression pattern between *S.* Typhimurium and *S.* Choleraesuis through 2-dimensional SDS-PAGE analysis^21^, indicating that only one enzyme, GabD, showed different expression level between the two serovars^21^. GabD is succinate semialdehyde dehydrogenase I and the expression level of GabD appeared not directly related to invasiveness of *S.* Choleraesuis. In this study we used proteomic approach, SILAC, to analyze the differential protein expression between *S.* Typhimurium and *S.* Choleraesuis. The two serovars shared 4021 proteins in common. A total of 1731 proteins were indentified, and more than 70% of the identified proteins did not express at different level between the two. Many flagella synthesis and chemotaxis response proteins were expressed higher in *S.* Typhimurium, suggesting that motility of *S.* Typhimurium may be better than *S.* Choleraesuis, as previously described^3^. Some proteins from predicted pseudogenes in *S.* Choleraesuis were also detected in our analysis. This may be because *Salmonella* pseudogenes could undertake a recoding mechanism^22^. Most of the genes expressed higher in *S.* Choleraesuis SC-B67 are metabolism-related genes. Interestingly many virulence factors, like those in SPI-1 and SPI-2, were higher expressed in *S.* Choleraesuis. The result might explain why *S.* Choleraesuis is more invasive than *S.* Typhimurium.

*Salmonella* encounter different acid stress environment from gastric acid (pH 2.0~3.0) to phagosome (pH 5.5~6.0) during its infection route^23^. Acid tolerance response is important for their virulence^24^. *S.* Choleraesuis was more tolerant to the SGF than *S.* Typhimurium. When *S.* Choleraesuis achieves the intestine lumen, they have to pass through the intestinal mucosal barrier before they establish systemic infection. In terms of the non-polar or polar epithelial cell invasion, *S.* Typhimurium and *S.* Choleraesuis were almost the same. However, all the *S.* Choleraesuis strains showed better penetration than the *S.* Typhimurium. Epithelial cell invasion indicates whether bacteria can entry the cells by their own ability. Monolayer cell penetration is more complicated than epithelial cell invasion. Besides entry into epithelial cells, bacteria have to have transcytose or disrupt tight junction to pass through the cellular barrier^25^,^26^. SPI-1- and SPI-4-encoded genes have been proved to play a vital role in invasion of epithelial cells^27^,^28^,^29^. These effectors also can disrupt the structure and function of tight junction, thereby helping *Salmonella* to penetrate intestine for further disseminatioin^29^,^30^. In our study, the SPI-1 and SPI-4 genes were shown highly expressed in *S.* Choleraesuis in SILAC data. High expression of these proteins might contribute to *S.* Choleraesuis to breach the intestinal barrier to further cause systemic infection.

After penetrating the intestine barrier, survival in the macrophage is an important issue for *Salmonella* to cause systemic infection. *S.* Choleraesuis showed better intra-macrophage survival than *S.* Typhimurium. SPI-2 T3SS and *spv* genes play a critical role in intracellular survival and systemic infection^31^,^32^,^33^. Our results showed that the level of proteins encoded in SPI-2 and *spv* was expressed at least 5-fold higher in *S.* Choleraesuis. This may explain why *S.* Choleraesuis had better intra-macrophage survival than *S.* Typhimurium.

HilA is a key positive regulator that directly binds the promoter of the structure and effector genes in SPI-1 T3SS^34^. HilA is negatively regulated by HilE which binds to HilD to inhibit the transcription of *hilA*^17^. In the genomic analysis of *S.* Choleraesuis, *hilE* was predicted as a pseudogene. We found in this study that the plasmid-harboring *S.* Typhimurium or *S*. Choleraesuis *hilE* ORF could restore the *hilE* function in *S.* Typhimurium *hilE* deletion strain, meaning that the HilE in *S.* Choleraesuis could still be functional. Recently Nuccio and Baumler analyzed different *Salmonella* serovars with their genome sequences available in NCBI^35^. They redefined some pseudogenes to normal genes, including *hilE* in *S.* Choleraesuis^35^. Combining the genomic and functional assays, the hyperexpression of SPI-1 T3SS in *S.* Choleraesuis found in this study appears not related to *hilE,* which previously was thought to be a pseudogene.

Environmental signals, such as oxygen, Mg^2+^, and osmolarity, also regulate the SPI-1 T3SS expression. High oxygen concentration is a negative regulator for *hilA* expression. High oxygen inhibits the expression of *hilD* which down regulates *hilA*^11^. We tested the sensitivity to oxygen of the two serovars. The expression of *hilA* and *hilD* in *S.* Choleraesuis was higher in high and low oxygen conditions (data not shown). Thus, oxygen appears not the factor that causes SPI-1 hyperexpression in *S.* Choleraesuis. On the other hand, PhoPQ TCS negatively regulates SPI-1 T3SS after sensing Mg^2+^ in the environment. When *Salmonella* invades into cells, it encounters a low pH and Mg^2+^ environment. Low Mg^2+^ activates the PhoPQ TCS to suppress SPI-1 T3SS^36^. However, under high Mg^2+^ condition, Mg^2+^ binds to PhoP, which in turn activates its phosphatase activity to remove the phosphate from autophosphorylated PhoP^37^. The expression of *hilA* and *hilD* showed significant difference in the two serovars under low Mg^2+^ concentration. Interestingly, *hilD* in *S.* Choleraesuis was expressed higher at normal and high Mg^2+^ concentrations. Moreover, the downstream gene, *pagC*, was less expressed in *S.* Choleraesuis. Taken together, *S.* Choleraesuis seemed to be more sensitive to Mg^2+^. High concentration of Mg^2+^ might cause more PhoP dephosphorylation, which subsequently inhibits the PhoPQ to suppress the *hilA* and *hilD*, or to activate the *pagC* in *S.* Choleraesuis. In conclusion, this study provides sufficient *in vitro* evidence to support that *S.* Choleraesuis is more invasive than *S*. Typhimuriunm by hyperexpressing T3SS virulence genes. These findings are consistent with the clinical observation that *S.* Choleraesuis is among the non-typhoid *Salmonella* serovars more invasive to the host.

## Materials and Methods

### Bacterial strains, plasmids, and mutagenesis

All *Salmonella* strains and plasmids used in this study are listed in Table S1. Bacteria were cultured on the Luria-Bartani (LB) agar at 37 °C, with added ampicillin (100 μg/ml), kanamycin (50 μg/ml), and tetracycline (12.5 μg/ml) when appropriate. A λ-red recombinase mutagenesis method was used for Δ*hilE* mutant construction, as previously described^38^. The primers used to amplify pKD4 kanamycin resistance gene are listed in Table S2. To construct the *hilE* complement strain, primers to amplify the *hilE*-contained the putative promoter region from the *S.* Typhimurium and *S.* Choleraesuis, respectively. PCR products were digested by *Bam*HI and *Eco*RI at 37 °C for 1 hour. Digested fragments were ligated into pBR322. The plasmids harbored the *hilE* from two serovars were then transferred to the Δ*hilE* mutant strains by electroporation.

### SILAC proteome analysis

To compare the protein expression pattern between *S.* Typhimuriuma LT2 and *S.* Choleraesuis SC-B67, we used the SILAC method as previously described^22^. Briefly, *S.* Typhimurium and *S.* Choleraesuis were cultured to late log phase in the SILAC medium (Mg^2+^, 0.4 mM) supplement with different isotope form of amino acids. Equal numbers of the two serovars were mixed together for further protein extraction by sonication. Protein concentration was measured by BCA method (Thermo). 40 μg of protein sample were separated by 2D-SCX/RPLC system (Dionex). Separated samples were analyzed by a LTQ-Orbitrap hybrid mass spectrometer (Thermo). Raw peptide sequences collected from the LTQ-Orbitrap were analyzed by Mascot v2.3 and MaxQuant v1.2 (Matrix Science). The sequences of *S.* Typhimurium (NC_003197) and *S.* Choleraesuis (NC_004631) were downloaded from NCBI and used as references for comparison. All experiments of SILAC analysis were repeated twice.

### qPCR validation

To test that the mRNA expression was consistent with SILAC results, *Salmonella* strains were cultured in SILAC media to late log-phase. Wild type, Δ*hilE,* and *hilE* complement strain of SL1344 were cultured in DMEM (Mg^2+^, 0.8 mM) or RPMI (Mg^2+^, 0.4 mM) media to late log-phase for analyzing the expression of *hilA* and *hilD*. To analyze the *hilD* expression under different oxygen level, SL1344 and SC-B67 were cultured in DMEM with (high oxygen) or without (low oxygen) shaking to late log phase. To compare the *hilD* and *pagC* expression level in different Mg^2+^ concentration, SL1344 and SC-B67 were inoculated in the N-salt media with 8 μM (low), 1 mM (normal), and 10 mM (high) of Mg^2+^ as previously described^39^. Bacteria RNA were isolated by TRIzol reagent (Invitrogen) according to the phenol-base method. RNA were treated with DNase I (Fermentas) 20 min at 37 °C to remove genomic DNA and then purified using a RNA clean up kit. Before converting RNA to cDNA, 1 μg of total RNA were used in PCR reaction to make sure that DNA has been removed. 1 μg of total RNA were reverse transcribed to cDNA by ToolsQuant II Fast RT kit (Tools Biotechnology Co., Ltd.). qPCR experiments were performed with SybrGreen Supermix (Bio-Rad) in an iCycler iQ5 (Bio-Rad) instrument. Expression of each gene was normalized to that of 16S rDNA. All the primers used in the experiments were listed in Table S2. All the qPCR experiments were done in triplicate in each independent experiment.

### Acid tolerance assay

*S.* Choleraesuis SC-B67 and *S.* Typhimurium SL1344 were precultured in LB broth and then 1 ml of the culture was transferred to the tube containing 4 ml of simulated gastric fluid (SGF, pH 2.0). Suspensions were cultured in the 37 °C incubator without shaking and survival was monitored every 5 mins. SGF contained 8.3 g/L proteose-peptone (Sigma), 3.5 g/L D-glucose (Sigma), 2.05 g/L NaCl (Sigma), 0.6 g/L KH_2_PO_4_ (Sigma), 0.11_ _g/L CaCl_2_ (Sigma), 0.37 g/L KCl (Sigma), 0.1 g/L lysozyme (Sigma), and 13.3 mg/L pepsin (Sigma)^40^. Final pH was adjusted to 2.0 with sterile 6.0 M HCl (Sigma). The recovered bacteria were counted by serial plating on LB agar at each time point with appropriate dilutions in PBS. Because the incubation period was short, experiment was done one group in each independent experiment. The experiment was repeated three times independently. We combined three independent experimental results for further statistical analysis.

### Invasion assay

MDCK cells were used to generate polarized epithelial cell monolayer. 1 × 10^6^ cells were cultured in 6-well plate for 5 days^29^. MDCK and HeLa cells, polar and non-polar, respectively, were infected by *S.* Thphimurium and *S*. Choleraesuis with MOI 100 as previously described^29^. At 1 hour post-infection, cells were washed with PBS and treated with gentamicin (75 μg/ml) for 30 mins. Cells were lysed by lysis buffer (0.5% Triton X-100 in PBS pH 7.4) and plated on LB agar with appropriate dilutions. Invasion experiments were done in triplicate in each independent experiment.

### Penetration assay

To measure the penetration ability of different strains of *Salmonella*, penetration assays were performed by using MDCK and Caco-2 cell monolayer with MOI 100, as previously described^41^. Penetrated *Salmonella* organisms were retrieved from the basolateral medium and were plated onto the LB agar media at 1, 3 and 6 hours after infection. *E. coli* RDEC-1, a non-invasive strain, was used as a negative control in every experiment to ensure the integrity of the cell monolayers^41^. Experiments were done in triplicate in each independent experiment.

### Intra-macrophage survival assay

Intra-macrophage survival of *Salmonella* in THP-1 cells was determined by a gentamicin protection assay, using methods described previously^9^. At 4 and 6 hours post-infection, intracellular bacteria were recovered with a lysis buffer (0.5% Triton X-100 in PBS pH 7.4) and were plated onto LB agar with or without appropriate antibiotics. Colonies were counted on the next day to calculate the intracellular survival rate. All the experiments were done in triplicate in each independent experiment.

### Mouse infection model and competitive index assay

Six- to eight-weeks-old female BALB/c mice were purchased from National Laboratory Animal Center, Taiwan. Mice were monitored daily during the experiments and sacrificed when it showed moribund or pain outcome. All the experiments were approved and followed the national animal care guidelines and the Institutional Animal Care and Use Committee (IACUC) of Chang Gung University (approval No CGU13–112). *Salmonella* were grown to the late-log phase. Bacteria were then washed twice and resuspensioned in saline. Six to ten mice were grouped and infected orally with *S.* Typhimurium or *S.* Choleraesuis (1 × 10^7^ cfu/mouse). Organs were harvested at 1-, 3-, and 5-day(s) post-infection and homogenized in saline. Bacteria were cultured on LB agar with appropriate dilutions. In competition assays, six- to eight-weeks-old female BALB/c mice were infected by *S.* Typhimurium and *S.* Choleraesuis mixed culture (2 × 10^7^ cfu/mouse). At 5 days post-infection, bacteria were recovered from the organs and plated onto LB agar media with or without chlorampnenicol (30μg/ml) to differentiate between *S.* Choleraesuis SC-B67 (resistant to chlorampnenicol), and *S.* Typhimurium SL1344 (susceptible to chlorampnenicol). Competitive Index (CI) is defined as the ratio between *S.* Choleraesuis and *S.* Typhimurium within output divided by the ration within the input.

### Statistical analysis

All the experiments were repeated three times. All the quantitative data in this study was performed mean ± standard error. The statistical analysis was calculated by the sigma plot (version 10.0). Unpaired t-test was used to compare the results in the study.

## Additional Information

**How to cite this article**: Huang, K.-Y. *et al.* Hyperinvasiveness of *Salmonella enterica* serovar Choleraesuis linked to hyperexpression of type III secretion systems *in vitro. Sci. Rep.* **6**, 37642; doi: 10.1038/srep37642 (2016).

**Publisher's note:** Springer Nature remains neutral with regard to jurisdictional claims in published maps and institutional affiliations.

## Supplementary Material

Supplementary Information

## Figures and Tables

**Table 1:** The expression level of virulence factors between *S.* Choleraesuis SC-B67 and *S.* Typhimurium LT2.

Virulence locus	ID	Gene	Ratio^a^
SPI-1	SC2794	*sitA*	0.99
	SC2802	*orgA*	4.66
	SC2806	*PrgH*	12.38
	SC2807	*hilD*	24.04
	SC2808	*hilA*	21.60
	SC2814	*sipA*	22.47
	SC2815	*sipD*	5.54
	SC2816	*sipC*	9.65
	SC2817	*sipB*	13.04
	SC2824	*invJ*	6.38
	SC2828	*invA*	17.86
SPI-2	SC1412	*ssrB*	9.77
	SC1415	*ssaC*	24.04
	SC1421	*sseC*	5.75
SPI-4	SC4140	*ssiE*	22.88
	SC4141	*ssiF*	5.04
SPI-5	SC1040	*pipB*	0.74
	SC1043	*sopB*	18.73
SPI-11	SC1256	*pagC*	3.63
SPI-13	SC3061	*stmR*	1.45
CS54	SC2510	*xseA*	0.84
	SC2513	*sinI*	2.26
Gifsy-1	SC2579	*gogB*	0.72
Gifsy-2	SC0997	*sodC*	1.45
	SC1459	*sodC*	4.69
pSLT	SCH_v26	*pefD*	0.57
	SCH-V05	*spvC*	129.87
	SCH_V04	*spvB*	45.25
	SCH_V48	*mig-5*	0.81
Regulators	SC1462	*slyA*	2.63
	SC3434	*ompR*	2.13
	SC4120	*zur*	1.76
	SC2896	*relA*	1.89
	SC1181	*phoQ*	1.26
	SC1182	*phoP*	1.17
	SC0713	*fur*	1.12
	SC1746	*hns*	1.26
	SC1748	*hnr*	1.51
	SC2645	*rpoE*	1.18
	SC2856	*rpoS*	1.85
	SC4014	*oxyR*	0.71
	SC3453	*rtcR*	1.27
	SC1950	*sirA*	1.01
Other effectors	SC2712	*virK*	5.60
	SC0798	*slrP*	7.42
	SC1852	*sopE2*	8.32
	SC0926	*sopD2*	15.38
	SC1174	*sifA*	4.66
	SC1626	*sseJ*	22.08
	SC1252	*msgA*	2.91
	SC2710	*pipB2*	6.75
	SC2713	*mig*	3.05
	SC4040	*sseK1*	6.76
	SC3130	*tolC*	1.10
	SC2708	*iroN*	0.41
	SC2290	*sseL*	6.38
	SC1691	*steC*	18.15

^a^SC-B67/LT2.
