# Supplementary material for: Hyperinvasiveness of Salmonella enterica serovar Choleraesuis linked to hyperexpression of type III secretion systems in vitro
Source: Sci Rep. 2016 Nov 25;6:37642. doi: 10.1038/srep37642 (PMC5122882; doi:10.1038/srep37642)
Supplement: Supplementary Information [file srep37642-s1.doc]

**Supplemental informations for**

**Hyperinvasiveness of *Salmonella enterica* serovar Choleraesuis linked to hyperexpression of type III secretion systems *in vitro***

Kuan-Yeh Huang^1,2^, Yi-Hsin Wang^2^, **Kun-Yi Chien^1^,** Rajendra Prasad Janapatla^2^ and Cheng-Hsun Chiu^1,2^

*^1^Graduate Institute of Biomedical Sciences, Chang Gung University College of Medicine, Taoyuan, Taiwan and ^2^Molecular Infectious Disease Research Center, Chang Gung Memorial Hospital, Chang Gung University College of Medicine, Taoyuan, Taiwan*

| Supplemental Table 1. *Salmonella* strains and plasmids used in this study | | | |
| --- | --- | --- | --- |
| Strains/plasmids | | | |
| Strains | | Characteristics | Source |
|  | LT2 | Wild-type, *S.* Typhimurium | 1 |
|  | SL1344 | Wild-type, *S.* Typhimurium | 2 |
|  | SC-B67 | Wild-type, *S.* Choleraesuis | 3 |
|  | RDEC-1 | Wild-type, *Escherichia coli* | 4 |
|  | ST145 | Wild-type, *S.* Typhimurium | This study |
|  | ST185 | Wild-type, *S.* Typhimurium | This study |
|  | SC-CY10 | Wild-type, *S.* Choleraesuis | This study |
|  | SC-CY18 | Wild-type, *S.* Choleraesuis | This study |
|  | SC-CY25 | Wild-type, *S.* Choleraesuis | This study |
|  | KY10 | Wild-type, pBR322 | This study |
|  | KY01 | SL1344, pKD46 | This study |
|  | KY02 | SL1344, *hilE^-^* | This study |
|  | KY02-Stm | SL1344, *hilE^-^*, pBR322-*hilE*-SL1344 | This study |
|  | KY02-Sc | SL1344, *hilE^-^*, pBR322-*hilE*-SC-B67 | This study |
| Plasmids | | | |
|  | pBR322 | Tc^R^, Ap^R^ | 5 |
|  | pKD46 | repA101(ts), araBp-gam-bet-exo | 6 |
|  | pKD3 | Kanamycin cassette | 6 |
|  | pHilE-Stm | HilE from *S.* Typhimurium in pBR322 | This study |
|  | pHilE-Sc | HilE from *S.* Choleraesuis in pBR322 | This study |

| Supplemental Table 2. Primers used in this study | |
| --- | --- |
| oligonucleotides | Sequences (5’ to 3’) |
| HilE-pKD3-F | AATCTGGTATACAGAGACACCAACGAAATGGTGTAGGCTGGAGCTGCTTC |
| HilE-pKD3-R | CTGCGAGTCCGCAAGCTTGTTTTGTCCTCACATATGAATATCCTCCTTA |
| 16SrDNA-qPCR-F | GAATGCCACGGTGAATACGTT |
| 16SrDNA-qPCR-R | ACCCACTCCCATGGTGTGA |
| HilA-qPCR-F | GTCCGGTCGTAGTGGTGTCT |
| HilA-qPCR-R | CGGCAGTTCTTCGTAATGGT |
| HilD-qPCR-F | ACTCGAGATACCGACGCAAC |
| HilD-qPCR-R | CTTCTGGCAGGAAAGTCAGG |
| SipB-qPCR-F-Stm | TCAGCTTCATCCAGTCGTCC |
| SipB-qPCR-R-Stm | GGATGCCGTTAGTGAAGGCG |
| SipB-qPCR-F-Sc | ATTGCCCTTCGCTGGAGAGT |
| SipB-qPCR-R-Sc | TGTGGTGGCAACGAAAGCGG |
| PagC-qPCR-F | CGGGTCTGTTGAGCCTGAAG |
| PagC-qPCR-R | TAGGCTGGCCCAACCATTAA |
| HilE-EcoRI-F | AAAGAATTCGGTTCTTATAGCGGCTATGG |
| HilE-BamHI-R | CCCGGATCCACTTGATGTGTATATAAAAAATGCC |

| Supplemental Table 3. Proteins with an expression level 2 times higher in *S.* Choleraesuis SC-B67 | | |
| --- | --- | --- |
| ID | Protein description | Ratio |
| SC2670 | YvrE; putative cytoplasmic protein | 232.56 |
| SCH_V05 | SpvC; 27.5 kDa virulence protein | 129.87 |
| SC3621 | Yin; putative mandelate racemase / muconate | 80.00 |
| SC3998 | KatG; Bacterial haem catalase/peroxidase | 60.98 |
| SC0113 | IlvI; acetolactate synthase III, valine sensitive, large subunit | 59.17 |
| SC3099 | YghA; putative oxidoreductase | 58.48 |
| SC3299 | YhcN; putative outer membrane protein | 51.81 |
| SC1042 | PipC; Pathogenicity island encoded protein: homologous to ipgE of Shigella | 49.50 |
| SC4191 | HilD; putative AraC-type DNA-binding domain-containing protein | 48.78 |
| SCH_V04 | SpvB; 65 kDa virulence protein | 45.25 |
| SC3083 | Kbp; putative ferrichrome-binding periplasmic protein | 35.21 |
| SC2093 | ManB; phosphomannomutase in colanic acid gene cluster | 33.11 |
| SC2801 | OrgB; putative flagellar biosynthesis/type III secretory pathway protein | 32.05 |
| SC0536 | UshA; UDP-sugar hydrolase 5'-nucleotidase | 31.95 |
| SC2822 | SpaB; surface presentation of antigens; secretory proteins | 31.85 |
| SC1418 | SseA; Secretion system effector SseA | 31.15 |
| SC0520 | putative transposase | 30.86 |
| SC4119 | YjbJ; putative cytoplasmic protein | 29.94 |
| SC3864 | MetE; 5-methyltetrahydropteroyltriglutamate- homocysteine S-methyltransferase | 28.82 |
| SC3954 | Sbp; ABC superfamily (bind_prot), sulfate transport protein | 26.46 |
| SC2553 | CadB; APC family, lysine/cadaverine transport protein | 26.39 |
| SC3975 | GlpK; glycerol kinase | 26.18 |
| SC1297 | YeaG; putative Ser protein kinase | 24.33 |
| SC1415 | SsaC; Secretion system apparatus SsaC | 24.04 |
| SC2807 | HilD; putative AraC-type DNA-binding domain-containing protein | 24.04 |
| SC4409 | OsmY; hyperosmotically inducible periplasmic protein, RpoS-dependent stationary phase gene | 23.98 |
| SC4215 | putative cytoplasmic protein | 23.04 |
| SC2818 | SicA; surface presentation of antigens; secretory proteins | 22.88 |
| SC4140 | SsiE; putative inner membrane protein | 22.88 |
| SC2814 | SipA; cell invasion protein | 22.47 |
| SC1626 | SseJ; translocated effector: regulated by SPI-2 | 22.08 |
| SC0758 | YbgS; putative homeobox protein | 22.03 |
| SC2808 | HilA; invasion genes transcription activator | 21.60 |
| SC2554 | CadA; lysine decarboxylase 1 | 21.46 |
| SC2156 | FbaB; 3-oxoacyl-[acyl-carrier-protein] synthase I | 19.53 |
| SC1043 | SopB; outer protein | 18.73 |
| SC1691 | SteC; putative inner membrane protein | 18.15 |
| SC2830 | InvG; invasion protein; outer membra | 18.05 |
| SC2100 | WcaM; putative colanic acid biosynthesis protein | 18.02 |
| SC2828 | InvA; invasion protein | 17.86 |
| SC3621 | putative mandelate racemase | 17.64 |
| SC1413 | SsrA; Secretion system regulator:Sensor component | 17.18 |
| SC3210 | YhbO; putative intracellular proteinase | 17.01 |
| SC1437 | SsaO; Secretion system apparatus SsaO | 16.56 |
| SC4138 | putative ABC exporter outer membrane component homolog | 16.29 |
| SC1227 | Gifsy-1 prophage minor tail protein | 16.23 |
| SC0755 | NadA; quinolinate synthetase, A protein | 15.60 |
| SC1725 | YciF; putative cytoplasmic protein | 15.60 |
| SC0926 | SopD2; homologous to secreted protein sopD | 15.38 |
| SC1936 | OtsB; trehalose-6-phosphate phophatase, biosynthetic | 15.20 |
| SC1218 | vg05; Gifsy-1 prophage head-tail preconnector gp5 | 14.81 |
| SC1510 | OpcC; putative periplasmic component, ABC transport system | 14.75 |
| SC2187 | YohF; putative oxidoreductase | 14.62 |
| SC1847 | YebV; putative cytoplasmic protein | 14.58 |
| SC1508 | OpbA; ABC-type proline/glycine betaine transport systems, ATPase component | 13.66 |
| SC1257 | putative periplasmic protein | 13.55 |
| SC2827 | InvB; surface presentation of antigens; secretory proteins | 13.33 |
| SC3093 | YghW; putative cytoplasmic protein | 13.23 |
| SC1349 | putative outer membrane protein | 13.21 |
| SC2923 | YgdI; putative lipoprotein | 13.16 |
| SC2817 | SipB; cell invasion protein | 13.04 |
| SC3828 | RffH; glucose-1-phosphate thymidylyltransferase | 12.85 |
| SC1339 | KatE; catalase; hydroperoxidase HPII(III), RpoS dependent | 12.42 |
| SC2805 | PrgI; cell invasion protein; cytoplasmic | 12.38 |
| SC2806 | PrgH; cell invasion protein | 12.38 |
| SC3467 | GlgX; glycosyl hydrolase | 12.08 |
| SC2727 | YgaU; putative LysM domain | 12.02 |
| SC2396 | PgtE; Phosphoglycerate transport: outer membrane protein E | 12.00 |
| SC2735 | YgaM; putative inner membrane protein | 11.85 |
| SC1564 | OsmC; putative resistance protein, osmotically inducible | 11.47 |
| SC1428 | SsaH; Secretion system apparatus SsaH | 11.39 |
| SC1509 | PsiF; induced by phosphate starvation | 11.17 |
| SC0425 | OpcB; putative binding-protein-dependent transport system, inner membrane component | 11.11 |
| SC4384 | YjiY; putative carbon starvation protein | 11.10 |
| SC2428 | CysK; subunit of cysteine synthase A and O-acetylserine sulfhydrolase A | 10.96 |
| SC0826 | Dps; stress response DNA-binding protein; starvation induced resistance to H_2_O_2_ | 10.92 |
| SC1882 | YobB; putative amidohydrolase | 10.89 |
| SC1436 | YobB; putative amidohydrolase | 10.80 |
| SC0407 | YahO; putative periplasmic protein | 10.72 |
| SC1100 | MsyB; acidic protein suppresses mutants lackingfunction of protein export | 10.38 |
| SC1935 | OtsA; trehalose-6-phosphate synthase | 10.20 |
| SC2832 | InvH;  invasion protein | 10.18 |
| SC0890 | PoxB; pyruvate dehydrogenase/oxidase FAD and thiamine PPi cofactors, cytoplasmic in absence of cofactors | 10.16 |
| SC1533 | YdeI; putative periplasmic protein | 10.07 |
| SC4137 | Mac; putative methyl-accepting chemotaxis protein | 10.04 |
| SC2311 | ElaB; putative inner membrane protein | 9.81 |
| SC0508 | YbaY; glycoprotein/polysaccharide metabolism | 9.80 |
| SC1412 | SsrB; Secretion system regulator: transcriptional activator | 9.77 |
| SC2816 | SipC; cell invasion protein | 9.65 |
| SC1568 | AdhP; alcohol dehydrogenase, propanol preferring | 9.43 |
| SC1433 | SsaL; Secretion system apparatus SsaL | 9.24 |
| SC1764 | ChaB; cation transport regulator | 9.11 |
| SCH_V49 | RlgA; integrase-like protein Y4LS | 8.93 |
| SC2646 | NadB; quinolinate synthetase, B protein | 8.51 |
| SC1852 | SopE2; TypeIII-secreted protein effector: invasion-associated protein | 8.32 |
| SC3683 | CigR; putative inner membrane protein | 7.91 |
| SC2181 | YehZ; putative ABC superfamily (bind_prot) transport protein (possibly glycine betaine choline transport for osmoprotection | 7.82 |
| SC1443 | SsaU; Secretion system apparatus SsaU | 7.79 |
| SC0169 | Gcd; glucose dehydrogenase | 7.70 |
| SC3991 | MetB; cystathionine gamma-synthase | 7.69 |
| SC0798 | SlrP; leucine-rich repeat protein | 7.42 |
| SC1435 | SsaV; Secretion system apparatus SsaV | 7.24 |
| SC3483 | UgpC; ABC superfamily (atp_bind), sn-glycerol 3-phosphate transport protein | 7.20 |
| SC0962 | PncB; nicotinate phosphoribosyltransferase | 6.93 |
| SC4040 | SseK1; putative cytoplasmic protein | 6.76 |
| SC2710 | PipB; Homolog of pipB, putative pentapeptide repeats | 6.75 |
| SC3449 | RtcA; RNA 3'-terminal phosphate cyclase | 6.64 |
| SC1266 | putative outer membrane lipoprotein | 6.62 |
| SC3301 | YhcO; putative cytoplasmic protein | 6.51 |
| SC2824 | InvJ; surface presentation of antigens; secretory proteins | 6.38 |
| SC2290 | SseL; putative cytoplasmic protein | 6.38 |
| SC1476 | RnfB; putative alternative beta subunit of Na+-transporting NADH:ubiquinone oxidoreductase | 6.17 |
| SC2829 | InvE; invasion protein | 6.01 |
| SC4360 | putative inner membrane or exported | 5.97 |
| SC2520 | GcpE; putative protein, involved in density-dependent regulation of peptidoglycan biosynthesis | 5.87 |
| SC3209 | YraR; putative nucleoside-diphosphate-sugar epimerase | 5.83 |
| SC1840 | KdgR; putative transcriptional repressor (IclR family) | 5.81 |
| SC2443 | CysP; ABC superfamily (bind_prot), thiosulfate transport protein | 5.78 |
| SC1421 | SseC; Secretion system effector SseC | 5.75 |
| SC1644 | YdbJ; putative cytoplasmic protein | 5.72 |
| SC1700 | OsmB; osmotically inducible lipoprotein | 5.67 |
| SC1226 | VmtV; Gifsy-1 prophage VmtV | 5.66 |
| SC2470 | TktB; transketolase 2, isozyme | 5.62 |
| SC2712 | VirK; virulence gene; homologous sequence to virK in Shigella | 5.60 |
| SC2469 | TlaA; transaldolase A | 5.55 |
| SC2815 | SipD; cell invasion protein | 5.54 |
| SC1532 | Ydej; putative Competence-damaged protein | 5.43 |
| SC1430 | SsaJ; Secretion system apparatus SsaJ | 5.41 |
| SC2271 | OmpC; outer membrane protein 1b (ib;c), porin | 5.37 |
| SC0732 | YbgK; putative carboxylase | 5.34 |
| SC1219 | HdpD; Gifsy-1 prophage head protein gpshp | 5.25 |
| SC1332 | OsmE; transcriptional activator of ntrL gene | 5.25 |
| SC1329 | Spy; periplasmic protein related to spheroblast formation | 5.11 |
| SC1351 | NucA; putative DNA/RNA non-specific endonuclease | 5.10 |
| SC0904 | YcaC; Homolog of slsA in *S.* Typhimurium | 5.07 |
| SC4141 | SsiF; putative ABC-type bacteriocin/lantibiotic exporter, contain an N-terminal double-glycine peptidase domain | 5.04 |
| SCH_V50 | hypothetical protein | 5.04 |
| SC2973 | PilT; putative nucleic acid-binding protein, contains PIN domain | 5.03 |
| SC3445 | MalQ; 4-alpha-glucanotransferase | 4.94 |
| SC3695 | BglA; putative glycosyl hydrolase family | 4.78 |
| SC2391 | YfcY; paral putative acetyl-CoA acetyltransferase | 4.72 |
| SC1459 | SodC; copper/zinc superoxide dismutase | 4.69 |
| SC0712 | NacR; putative transcriptional regulator, LysR family | 4.69 |
| SC2802 | OrgA; putative inner membrane protein | 4.66 |
| SC1174 | SifA; lysosomal glycoprotein (lgp)-containing structures; replication in macrophages | 4.66 |
| SC0511 | RpmE2; putative 50S ribosomal protein L31 (second copy) | 4.56 |
| SC0377 | Deh; putative hydrolase or acyltransferase | 4.52 |
| SC3486 | UgpB; ABC superfamily (peri_perm), sn-glycerol 3-phosphate transport protein | 4.52 |
| SC3537 | TreF; cytoplasmic trehalase | 4.44 |
| SC3605 | putative transcriptional regulator | 4.35 |
| SC3727 | YidH; putative inner membrane protein | 4.33 |
| SC1341 | YajN; part of a kinase, putative domain shared with transporter | 4.31 |
| SC1301 | YeaD; putative enzymes related to aldose 1-epimerase | 4.28 |
| SC2724 | GabT; 4-aminobutyrate aminotransferase | 4.22 |
| SC3863 | MetR; regulator for metE and metH (LysR family) | 4.20 |
| SC2131 | YegO; putative resistance protein (efflux transporter), outer membrane | 4.14 |
| SC0076 | putative secreted protein | 4.06 |
| SC3109 | YghE; 2,5-diketo-D-gluconate reductase A | 4.01 |
| SC1710 | CysB; transcriptional regulator for cysteine regulon (LysR familiy) | 4.00 |
| SC0077 | putative secreted protein | 3.99 |
| SC1900 | ZnuC; ABC superfamily (atp_bind) high affinity Zn transport protein | 3.96 |
| SC1296 | YeaH; putative cytoplasmic protein | 3.95 |
| SC1566 | RpsV; 30S ribosomal subunit protein S22 | 3.93 |
| SC3793 | Kut; KUP family, potassium transport system, low affinity | 3.92 |
| SC3450 | RtcB; putative cytoplasmic protein | 3.92 |
| SC1262 | Ibp; putative molecular chaperone (small heat shock protein) | 3.88 |
| SC0756 | Pnuc; NMN family, nucleoside/purine/pyrimidine transporter | 3.88 |
| SC1660 | YdcN; putative transcriptional regulator | 3.83 |
| SC4222 | FrdA; fumarate reductase, anaerobic, flavoprotein subunit | 3.81 |
| SC2997 | putative monooxygenase | 3.79 |
| SC0447 | Tgt; tRNA-guanine transglycosylase | 3.78 |
| SC1620 | YdcI; putative transcriptional regulators, LysR family | 3.75 |
| SC0783 | YbhE; putative 3-carboxymuconate cyclase | 3.72 |
| SC1274 | YodA; putative periplasmic protein | 3.70 |
| SC1335 | CelC; PTS family, sugar specific enzyme III for cellobiose, arbutin, and salicin | 3.70 |
| SC0251 | YafB; 2,5-diketo-D-gluconate reductase B | 3.65 |
| SC2952 | LysA; diaminopimelate decarboxylase | 3.65 |
| SC1256 | PagC; Virulence-related outer membrane protein | 3.63 |
| SC2408 | DciP; putative thiamine pyrophosphate enzymes | 3.61 |
| SC2397 | PgtA; Phosphoglycerate transport: activator | 3.60 |
| SC0387 | putative response regulator | 3.59 |
| SC4221 | FrdB; fumarate reductase, anaerobic, Fe-S protein subunit | 3.56 |
| SC3060 | CliB; putative transcriptional regulator, LysR family | 3.55 |
| SCH_V51 | hypothetical protein | 3.52 |
| SC1586 | YncB; putative NADP-dependent oxidoreductase | 3.51 |
| SC4420 | LplA; lipoate-protein ligase A | 3.51 |
| SC0956 | OmpF; outer membrane protein 1a (ia;b;f), porin | 3.51 |
| SC4062 | AceB; malate synthase A | 3.42 |
| SC3087 | HybD; putative processing element for hydrogenase-2 | 3.40 |
| SC0515 | Hha; hemolysin expression modulating protein (involved in environmental regulation of virulence factors) | 3.40 |
| SC1446 | Cfa; cyclopropane fatty acyl phospholipid synthase | 3.39 |
| SC4063 | AceA; malate synthase A | 3.38 |
| SC1910 | YecD; putative isochorismatase | 3.35 |
| SC2962 | YohL; putative cytoplasmic protein | 3.34 |
| SC2474 | YfeW; putative beta-lactamase class C | 3.34 |
| SC0075 | YgdI; putative outer membrane lipoprotein | 3.32 |
| SC1707 | AcnA; aconitate hydratase 1 | 3.29 |
| SC1735 | Cls; cardiolipin synthase | 3.27 |
| SC3468 | GlgB; 1,4-alpha-glucan branching enzyme | 3.27 |
| SCH_V51 | hypothetical prot | 3.27 |
| SC4165 | LpxO; putative dioxygenase for synthesis of lipid | 3.23 |
| SC2803 | PrgK; cell invasion protein; lipoprotein, may link inner and outer membranes | 3.18 |
| SC3346 | mechanosensitive channel | 3.17 |
| SC3465 | GlgA; glycogen synthase | 3.16 |
| SC2497 | PurN; polyphosphate kinase, component of RNA degradosome | 3.11 |
| SC0106 | LeuD; 3-isopropylmalate isomerase (dehydratase), subunit with LeuC | 3.05 |
| SC2713 | Mig; putative transcription activator | 3.05 |
| SC1483 | YdgA; putative periplasmic protein | 3.00 |
| SC1899 | ZnuA; ABC superfamily (bind_prot) high affinity Zn transport protein | 2.96 |
| SC2449 | AmiA; N-acetylmuramoyl-l-alanine amidase I | 2.95 |
| SC3466 | GlgC: glucose-1-phosphate adenylyltransferase | 2.94 |
| SC1252 | MsgA; Macrophage survival gene; reduced mouse virulence | 2.91 |
| SC0180 | PanD; aspartate 1-decarboxylase | 2.86 |
| SC2390 | YfcX; paral putative dehydrogenase | 2.83 |
| SC0856 | YliJ; putative glutathione S-transferase | 2.82 |
| SC0682 | YbeL; putative cytoplasmic protein | 2.79 |
| SC1368 | Pps; phosphoenolpyruvate synthase | 2.77 |
| SC3480 | Ggt; gamma-glutamyltranspeptidase | 2.76 |
| SC1068 | YccJ; putative cytoplasmic protein | 2.74 |
| SC0424 | YaiB; putative cytoplasmic protein | 2.73 |
| SC3088 | HybC; hydrogenase-2, large subunit | 2.71 |
| SC2286 | GlpT; MFS family, sn-glycerol-3-phosphate transport protein | 2.71 |
| SC3618 | LldD; L-lactate dehydrogenase | 2.68 |
| SC1462 | SlyA; transcriptional regulator for hemolysin (MarR family) | 2.63 |
| SC0430 | YaiA; putative cytoplasmic protein | 2.63 |
| SC1657 | YdaA; putative universal stress protein | 2.62 |
| SC3464 | GlgP; glycogen phosphorylase | 2.61 |
| SC3308 | CafA; RNase G | 2.59 |
| SC4061 | MetA; homoserine transsuccinylase | 2.59 |
| SC2826 | InvC; surface presentation of antigens; secretory proteins | 2.57 |
| SC3751 | DgoA; putative mandelate racemase / muconate lactonizing enzyme family | 2.57 |
| SC3176 | YgjD; putative inner membrane protein | 2.56 |
| SC3967 | YneB; putative fructose-1,6-bisphosphate aldolase | 2.53 |
| SC0870 | PotF; ABC superfamily (peri_perm), putrescine transporter | 2.51 |
| SC3966 | YneA; putative ABC superfamily (peri_perm), sugar transport protein | 2.47 |
| SC1116 | YceH; putative cytoplasmic protein | 2.46 |
| SC3137 | DsbA; putative thiol-disulfide isomerase and thioredoxin | 2.45 |
| SC0455 | YajI; putative outer membrane lipoprotein | 2.45 |
| SC1452 | YdhD; putative glutaredoxin protein | 2.43 |
| SC3330 | YhdV; putative outer membrane lipoprotein | 2.40 |
| SC3642 | RfaB; UDP-D-galactose: (glucosyl)lipopolysaccharide-1, 6-D-galactosyltransferase | 2.38 |
| SC2556 | GlnB; Nitrogen regulatory protein P-II 1 | 2.38 |
| SC3009 | YggB; putative membrane protein, involved in stability of MscS mechanosensitive channel | 2.37 |
| SC4069 | PepE; (alpha)-aspartyl dipeptid | 2.37 |
| SC2975 | putative periplasmic protein | 2.34 |
| SC3297 | Mdh; malate dehydrogenase | 2.32 |
| SC1799 | NhaB; NhaB family of transport protein, Na+/H+ antiporter, regulator of intracellular pH | 2.31 |
| SC0864 | YbjL; putative transport protein | 2.31 |
| SC2228 | YeiR; putative cobalamin synthesis protein | 2.31 |
| SC1795 | DadX; alanine racemase 2, catabolic | 2.28 |
| SC2513 | SinI; putative outer membrane protein | 2.26 |
| SC2994 | GcvP; glycine cleavage complex protein P, glycine decarboxylase | 2.25 |
| SC2089 | WzzB; regulator of length of O-antigen component of lipopolysaccharide chains | 2.24 |
| SC1674 | YcjG; putative chloromuconate cycloisomerase (muconate cycloisomerase) | 2.23 |
| SC3426 | YrfE; putative NTP pyrophosphohydrolase | 2.23 |
| SC2998 | UbiH; 2-octaprenyl-6-methoxyphynol hydroxylase | 2.21 |
| SC3851 | CorA; MIT family, Mg2+/Ni2+/Co2+ transport protein (Mg transport system I) | 2.18 |
| SC2099 | GalF; putative glucose-1-phosphate uridylyltransferase (UDP-glucose pyrophosphorylase), non-catalytic subunit | 2.18 |
| SC0543 | YbbL; putative ABC-type sugar/spermidine/putrescine transport system, ATPase component | 2.17 |
| SC2270 | ApbE; putative thiamine biosynthesis lipoprotein | 2.17 |
| SC3248 | YrbC; putative ABC superfamily (atp&memb), transport protein | 2.17 |
| SC1450 | SodB; superoxide dismutase, iron | 2.17 |
| SC2546 | putative periplasmic or exported protein | 2.16 |
| SC1471 | Nth; endonucleaseIII; DNA glycosylase/apyrimidinic lyase | 2.16 |
| SC1820 | SdaA; L-serine deaminase I/L-threonine deaminase I | 2.15 |
| SC2934 | RecC; exonuclease V, subunit | 2.15 |
| SC0211 | YaeH; putative cytoplasmic protein | 2.14 |
| SC3734 | YidR; putative cytoplasmic protein | 2.13 |
| SC3434 | OmpR; Transcriptional regulatory protein ompR | 2.13 |
| SC0413 | HemB; 5-aminolevulinate dehydratase | 2.12 |
| SC1112 | YceB; putative outer membrane lipoprotein | 2.12 |
| SC2471 | putative cytoplasmic protein | 2.11 |
| SC2251 | NerP; response regulator in two-component regulatory system with NarQ | 2.11 |
| SC3392 | YheR; putative NAD(P)H oxidoreductase | 2.10 |
| SC1808 | mind; cell division inhibitor | 2.08 |
| SC3030 | MetK; methionine adenosyltransferase 1 (AdoMet synthetase | 2.08 |
| SC4072 | YjbC; putative pseudouridine synthase | 2.08 |
| SC0157 | AcnB; aconitate hydratase 2 | 2.07 |
| SC2995 | GcvH; glycine cleavage complex protein H, carrier of aminomethyl moiety via covalently bound lipoyl cofactor | 2.06 |
| SC0044 | putative nitrite reductase | 2.06 |
| SC3613 | YibL; putative cytoplasmic protein | 2.06 |
| SC3536 | YurK; putative regulatory protein, gntR family | 2.06 |
| SC0103 | YabN; paral putative periplasmic binding protein of transport system | 2.04 |
| SC0517 | AcrB; RND family, acridine efflux pump | 2.02 |
| SC3159 | Mug; DNA glycosylase, G/U mismatch specific | 2.01 |
| SC3077 | UxuB; putative D-mannonate oxidoreductase | 2.01 |

| Supplemental Table 4. Proteins with an expression level 2 times higher in *S.* Typhimurium LT2 | | |
| --- | --- | --- |
| ID | Protein description | Ratio |
| SC1469 | Gst; glutathionine S-transferase | 37.69 |
| SC1604 | YdcL; putative outer membrane lipoprotein | 37.47 |
| SC1529 | Dcp; dipeptidyl carboxypeptidase II | 29.00 |
| SC4230 | ArtJ; putative arginine-binding periplasmic protein | 27.39 |
| STM0874 | MdaA(pseudo) | 24.72 |
| SC0140 | GuaC; GMP reductase | 22.52 |
| SC1714 | YciK; putative oxoacyl-(acyl carrier protein) reductase | 18.69 |
| STM1147 | putative ACR protein(pseudo) | 18.61 |
| STM2532 | putative inner membrane lipoprotein(pseudo) | 17.49 |
| STM2194 | YeiG(pseudo) | 17.37 |
| SC3550 | YhiL; putative TPR-repeat-containing protein | 16.37 |
| SC1672 | MppA; periplasmic murein tripeptide transport protein, also negative regulator of mulitple antibiotic resistance | 16.26 |
| SC3515 | YhiH; putative ABC-type multidrug transport system, ATPase component; Permease component of an ABC-transporter | 15.53 |
| SC4000 | GldA; glycerol dehydrogenase, NAD | 14.37 |
| STM2771 | FljB(pseudo) | 14.28 |
| SC2300 | PmrF; putative glycosyl transferase | 13.14 |
| STM1989 | YedA(pseudo) | 12.91 |
| STM0617 | Rna(pseudo) | 12.81 |
| SC1587 | YncA; putative acyltransferase | 12.56 |
| STM1062 | Uup(pseudo) | 12.50 |
| SC1410 | putative inner membrane protein | 11.85 |
| SC1528 | YdfG; putative oxidoreductase | 11.69 |
| SC2355 | HisJ; ABC superfamily (bind_prot), histidine transport protein | 11.59 |
| STM4519 | putative NAD-dependent aldehyde dehydrogenase(pseudo) | 11.55 |
| SC0432 | YaiE; putative cytoplasmic protein | 10.41 |
| SC2299 | YfbE; putative DegT/DnrJ/EryC1/StrS family | 10.29 |
| SC0663 | YbeF; putative transcriptional regulator, LysR family | 9.23 |
| SC3444 | GntT; GntP family, high-affinity gluconate permease in GNT I system | 9.10 |
| STM1746.S | OppA; oligopeptide transport protein | 9.02 |
| STM0826 | ybin(pseudo) | 8.67 |
| SC3001 | YgfE; putative cytoplasmic protein | 8.48 |
| SC3336 | YrdB; putative periplasmic protein | 8.42 |
| SC0473 | PhnX; 2-aminoethylphosphonate transport | 8.10 |
| SC1742 | OppA; ABC superfamily (periplasm), oligopeptide transport protein with chaperone properties | 7.96 |
| SC2884 | YgcF; putative Organic radical activating enzymes | 7.96 |
| SC2385 | MepA; murein DD-endopeptidase, penicillin-insensitive | 7.72 |
| SC2485 | GcvR; transcriptional repressor of gcv operon | 7.68 |
| SC1999 | CspB; putative cold-shock protein | 7.42 |
| STM1169 | MviM(pseudo) | 7.34 |
| SC1069 | WraB; trp-repressor binding protein | 7.29 |
| SC3254 | YrbI; putative protein of HAD superfamily, CMP-Neu5Ac homologs | 7.09 |
| STM3295 | FolP(pseudo) | 6.65 |
| SC2744 | ProW; ABC superfamily (membrane), glycine/betaine/proline transport protein | 6.49 |
| SC1324 | Xtha; exonuclease III, may repair singlet oxygen induced lesions | 6.36 |
| SC2912 | FucO; L-1,2-propanediol oxidoreductase | 6.32 |
| SC1791 | YcgR; putative inner membrane protein | 6.30 |
| SC3581 | CspA; Cold shock protein cspA (CSP-A) | 5.84 |
| SC4328 | YtgA; putative inner membrane protein | 5.81 |
| SC2330 | IrhA; NADH dehydrogenase transcriptional repressor (LysR family) | 5.53 |
| SC1741 | OppB; ABC superfamily (membrane), oligopeptide transport protein | 5.42 |
| SCH_V36 | TraT complement resistance protein precursor | 5.39 |
| SC1885 | YebE; putative inner membrane protein | 5.27 |
| STM1462.S | YdgJ(pseudo) | 5.11 |
| SC4301 | Mir; putative selenocysteine synthase [L-seryl-tRNA(Ser) selenium transferase | 4.98 |
| SC2393 | FadL; transport of long-chain fatty acids; sensitivity to phage T2 | 4.90 |
| SC1739 | OppD; ABC superfamily, oligopeptide transport protein | 4.79 |
| SC2381 | YfcK; putative peptidase | 4.78 |
| SC3408 | NirB; nitrite reductase, large subunit | 4.77 |
| SC2298 | Ais; aluminum inducible protein | 4.70 |
| SC2676 | RimM; 16S rRNA processing protein | 4.65 |
| SC0500 | putative cysteine synthase/cystathionine beta-synthase | 4.52 |
| SC2743 | ProV; ABC superfamily, glycine/betaine/proline transport protein | 4.43 |
| SC2707 | IroE | 4.31 |
| STM2299 | YfbG(pseudo) | 4.30 |
| SC3079 | Mac; putative methyl-accepting chemotaxis protein | 4.26 |
| SC4171 | BasR; response regulator in two-component regulatory system with BasS | 4.17 |
| SC1030 | YccU; putative cytoplasmic protein | 4.16 |
| SC3576 | biotin sulfoxide reductase | 4.16 |
| SC1591 | SrfB; ssrAB activated gene | 4.07 |
| SC1127 | FlgH; flagellar biosynthesis, basal-body outer-membrane L (lipopolysaccharide layer) ring protein | 3.98 |
| SC1126 | FlgG; flagellar biosynthesis, cell-distal portion of basal-body rod | 3.73 |
| SC1026 | YccF; putative inner membrane protein | 3.62 |
| SC2745 | ProX; ABC superfamily (bind_prot), glycine/betaine/proline transport protein | 3.57 |
| SC0002 | ThrA; aspartokinase I | 3.54 |
| SC1590 | SrfA; ssrAB activated gene | 3.51 |
| SC4170 | BasS; sensory kinase in two-component regulatory system with BasR | 3.48 |
| SC2090 | Udg; UDP-glucose/GDP-mannose dehydrogenase | 3.46 |
| SC1975 | FliG; flagellar biosynthesis, component of motor switching and energizing | 3.40 |
| SC0453 | YajD; putative cytoplasmic protein | 3.29 |
| SC2477 | YffB; putative glutaredoxin family | 3.26 |
| SC1927 | CheW; purine-binding chemotaxis protein; regulation | 3.25 |
| SC0131 | LpxC; UDP-3-O-acyl N-acetylglucosamine deacetylase | 3.25 |
| SC1718 | TrpH; trpR controlled transcriptional unit in the 5' upstream region of the trp operon | 3.24 |
| SC0003 | ThrB; homoserine kinase | 3.23 |
| SC1738 | OppF; ABC superfamily (ATP-binding), oligopeptide transport protein | 3.20 |
| SC4128 | AphA non-specific acidp hosphatase/phosphotransferase, class B | 3.19 |
| SC1940 | YecR; putative outer membrane lipoprotein | 3.19 |
| SC1977 | FliI; flagellum-specific ATP synthase | 3.17 |
| SC1164 | YcfR; putative outer membrane protein | 3.13 |
| SC4385 | Tsr; methyl-accepting chemotaxis protein I, serine sensor receptor | 3.09 |
| SC1103 | YceA; putative enzyme related to sulfurtransferases | 3.06 |
| SC1907 | YebC; putative cytoplasmic protein | 3.02 |
| SC1980 | FliL; flagellar biosynthesis | 3.01 |
| SC4407 | YjjG; putative haloacid dehalogenase-like hydrolase | 2.95 |
| SC4168 | PhnA; putative alkylphosphonate uptake protein in phosphonate metabolism | 2.95 |
| SC0139 | YacE; putative nucleotide kinase | 2.88 |
| SC1621 | Trg; methyl-accepting chemotaxis protein III, ribose and galactose sensor receptor | 2.82 |
| SC1920 | FlhA; flagellar biosynthesis; possible export of flagellar proteins | 2.76 |
| SC4127 | TyrB; tyrosine aminotransferase, tyrosine repressible | 2.76 |
| SC3694 | putative inner membrane protein | 2.73 |
| SC3171 | YgjT; putative resistance protein | 2.71 |
| SC0795 | BioD; dethiobiotin synthetase | 2.67 |
| SC3506 | Tcp; methyl-accepting transmembrane citrate/phenol chemoreceptor | 2.67 |
| SC1976 | FliH; flagellar biosynthesis; possible export of flagellar proteins | 2.67 |
| SC2227 | YeiP; putative elongation factor | 2.65 |
| SC2647 | YfiC; putative aminopeptidase | 2.64 |
| SC1165 | YcfS; putative periplasmic protein | 2.61 |
| SC1559 | putative monooxygenase | 2.60 |
| SC1929 | MotB; sensory histitine protein kinase, transduces signal between chemo- signal receptors and CheB and CheY | 2.58 |
| SC4278 | CpdB; 2':3'-cyclic-nucleotide 2'-phosphodiesterase | 2.47 |
| SC3648 | KdtB; phosphopantetheine adenylyltransferase | 2.46 |
| SC1928 | CheA; sensory histitine protein kinase, transduces signal between chemo- signal receptors and CheB and CheY | 2.43 |
| SC2708 | IroN; TonB-dependent siderophore receptor protein | 2.42 |
| SC0004 | ThrC; threonine synthase | 2.40 |
| SC1124 | FlgE; flagellar biosynthesis, hook protein | 2.39 |
| SC1635 | YdcF; putative inner membrane protein | 2.38 |
| SC0779 | ModA; ABC superfamily, molybdate transporter | 2.36 |
| SC0528 | HtpG; chaperone Hsp90, heat shock protein C 625 | 2.36 |
| STM4176 | PurH(pseudo) | 2.35 |
| SC3931 | putative branched-chain amino acid permease | 2.35 |
| SC1888 | PurT; phosphoribosylglycinamide formyltransferase 2 | 2.32 |
| SC1522 | RpsA; putative dehydratase, starvation sensing protein | 2.31 |
| SC1774 | PrsA; phosphoribosylpyrophosphate synthetase | 2.30 |
| SC2531 | PepB; putative aminopeptidase | 2.30 |
| SC3443 | YhgI; putative Thioredoxin-like proteins and domain | 2.26 |
| STM0477 | AcrR(pseudo) | 2.26 |
| SC0959 | DpaL; putatiave diaminopropionate ammonia lyase | 2.24 |
| SC4393 | MdoB; phosphoglycerol transferase I | 2.24 |
| SC0163 | PdxA; pyridoxine phosphate biosynthetic protein | 2.22 |
| SC1634 | YbcY; putative SAM-dependent methyltransferases | 2.22 |
| SC0165 | SpeD; S-adenosylmethionine decarboxylase, proenzyme | 2.20 |
| SC3098 | putative cytoplasmic protein | 2.20 |
| SC2314 | CheV; putative chemotaxis signal transduction protein | 2.19 |
| SC4071 | YaiL; putative cytoplasmic protein | 2.19 |
| SC2382 | YfcL; putative cytoplasmic protein | 2.18 |
| SC2476 | AcrD; RND family, aminoglycoside/multidrug efflux pump | 2.17 |
| SC3284 | YhcM; putative ATPase | 2.16 |
| SC3097 | putative cytoplasmic protein | 2.15 |
| SC3162 | Mcp2; putative methyl-accepting chemotaxis protein | 2.14 |
| SC2215 | CirA; outer membrane porin, receptor for colicin I, requires TonB | 2.13 |
| SC0213 | DapD; 2,3,4,5-tetrahydropyridine-2-carboxylate N-succinyltransferase | 2.13 |
| SC0817 | RhlE; putative ATP-dependent RNA helicase | 2.12 |
| SC1922 | CheZ; chemotactic response; CheY protein phophatase | 2.09 |
| SC2810 | SptP; protein tyrosine phosphate | 2.08 |
| SC1923 | CheY; chemotaxis regulator, transmits chemoreceptor signals to flagelllar motor components | 2.07 |
| SC3317 | AccB; acetylCoA carboxylase, BCCP subunit, carrier of biotin | 2.07 |
| SC3272 | CodA; putative cytosine deaminase | 2.07 |
| SC4056 | PurD; phosphoribosylglycinamide synthetase (GAR synthetase) | 2.05 |
| SC4314 | PyrI; aspartate carbamoyltransferase, regulatory subunit (allosteric regulation) | 2.05 |
| SC2900 | GudD; d-glucarate dehydratase | 2.04 |
| SC3538 | putative inner membrane protein | 2.03 |
| SC2151 | YegQ; putative protease | 2.02 |
| SC0629 | EntA; 2,3-dihydro-2,3-dihydroxybenzoate dehydrogenase | 2.01 |

A.


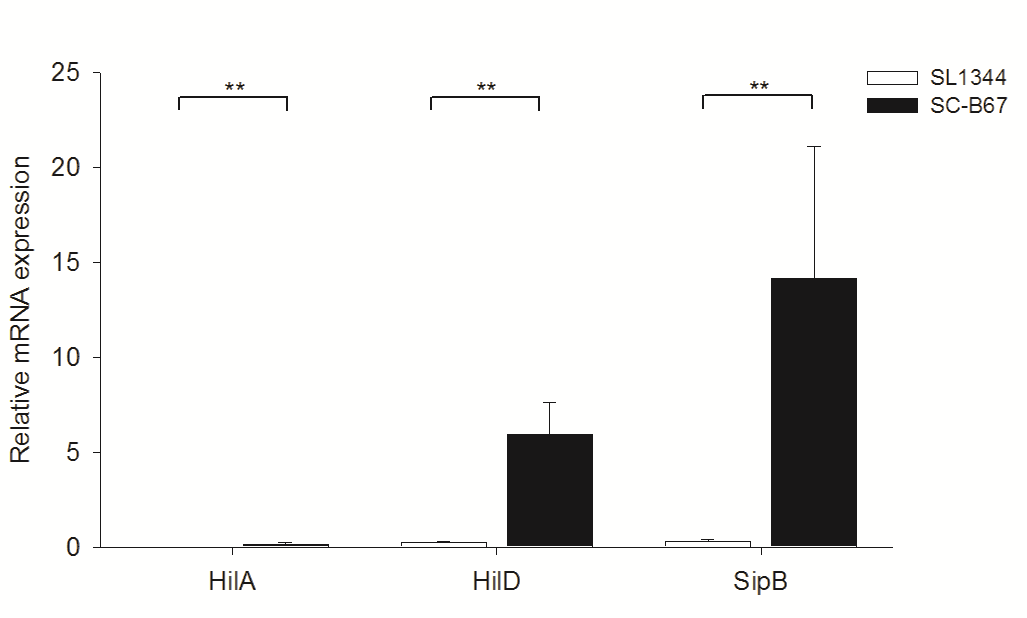


B.


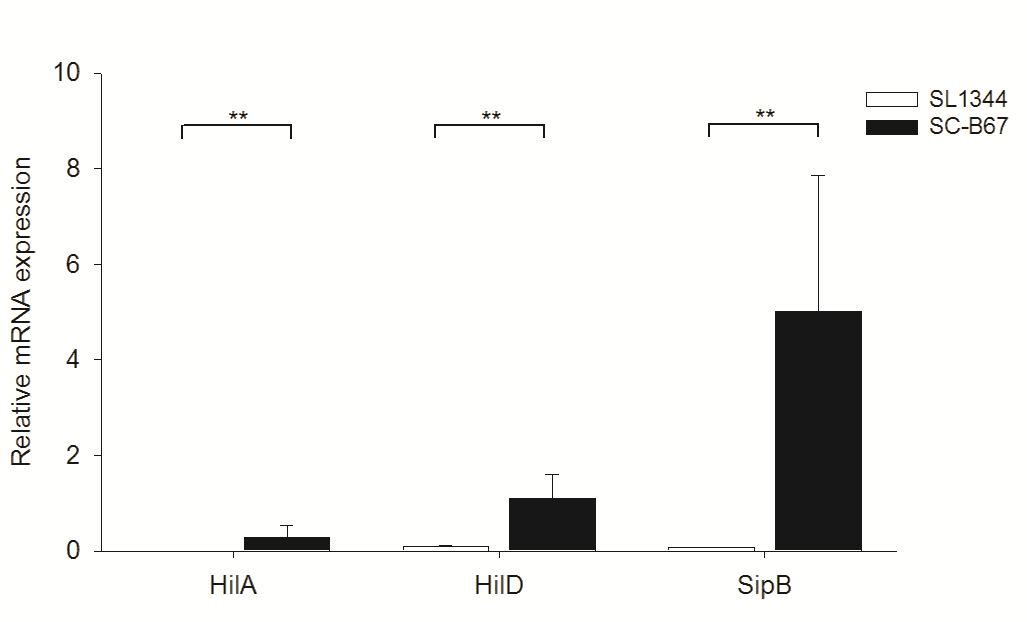


**Supplemental Figure 1.** Expression level of *hilA*, *hilD*, and *sipB* in *S*. Typhimurium SL1344 and *S*. Choleraesuis SC-B67 in cell culture medium. Both strains were incubated in DMEM (A) and RPMI (B) before detection. Expression level of each gene was normalized with 16S rDNA expression level. **, p<0.01


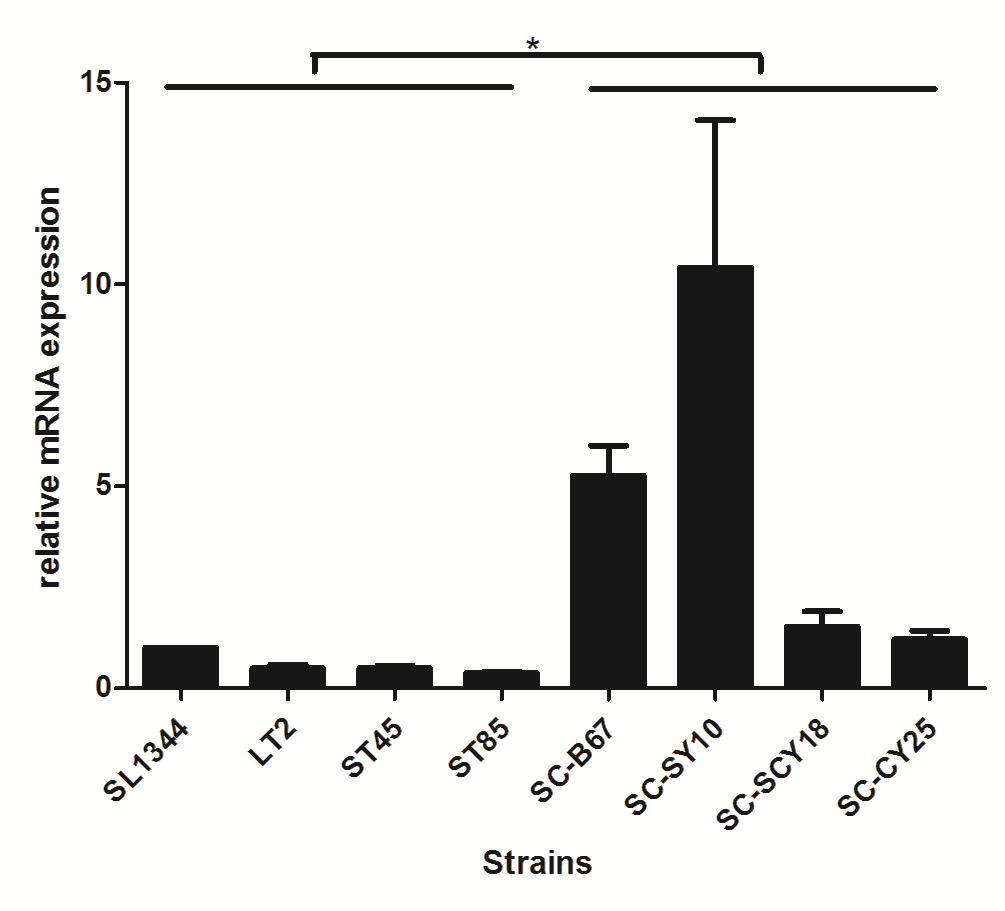


**Supplemental Figure 2.** Expression of *hilD* in SC-B67 and 3 clinical *S.* Choleraesuis isolates is compared to that of SL1344, LT2, and 2 clinical *S.* Typhimurium isolates. All the bacterial strains were cultured in DMEM for 6 hours prior to the experiment. The total RNA were isolated and analyzed by qPCR. All the expression level were relative to the 16S rDNA. *, p<0.05

**
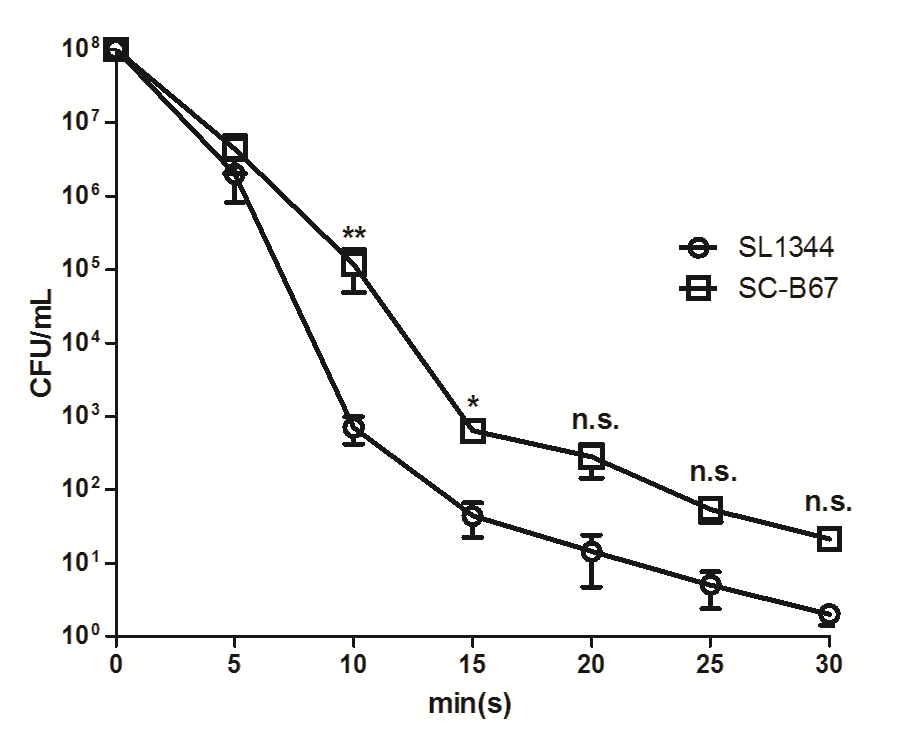
**

**Supplemental Figure 3.** Acid tolerance assay of *S*. Typhimurium SL1344 and *S*. Choleraesuis SC-B67. Both strains were cultured for 24 hours first and added into SGF at a 1:10 ratio. At each time point, the bacterial count was calculated by plating the bacterial culture on LB agar. *, p<0.05. **, p<0.01. n.s., no significance.

A.


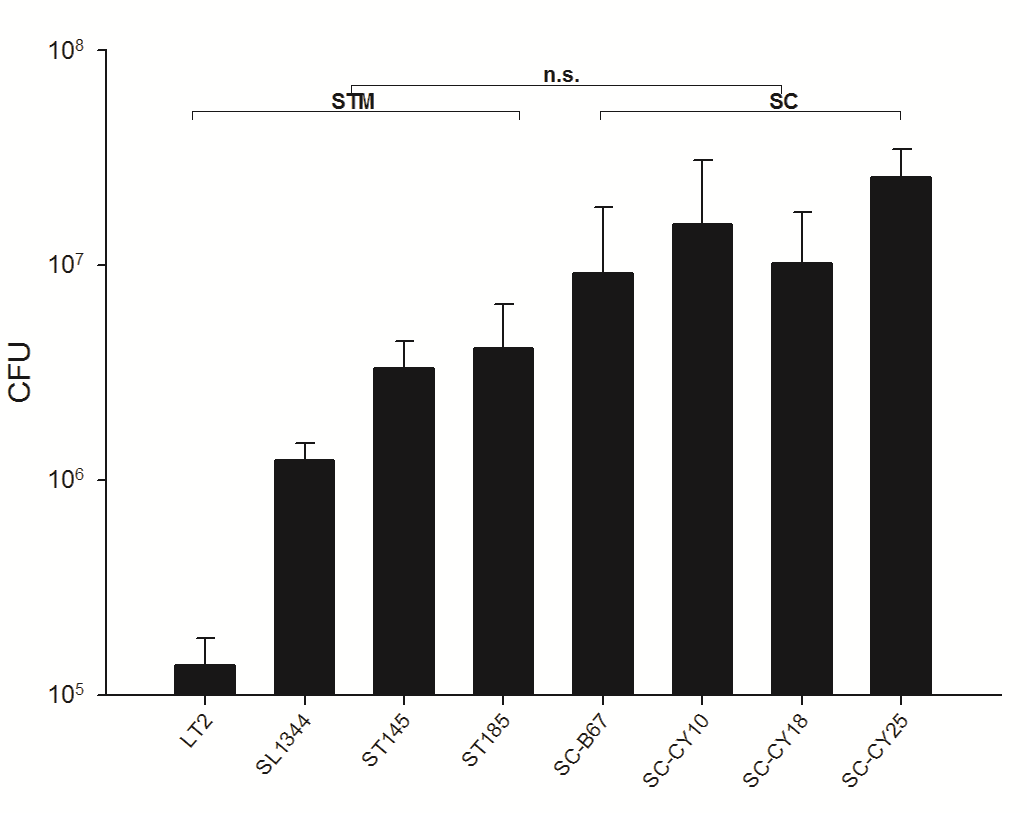


B.


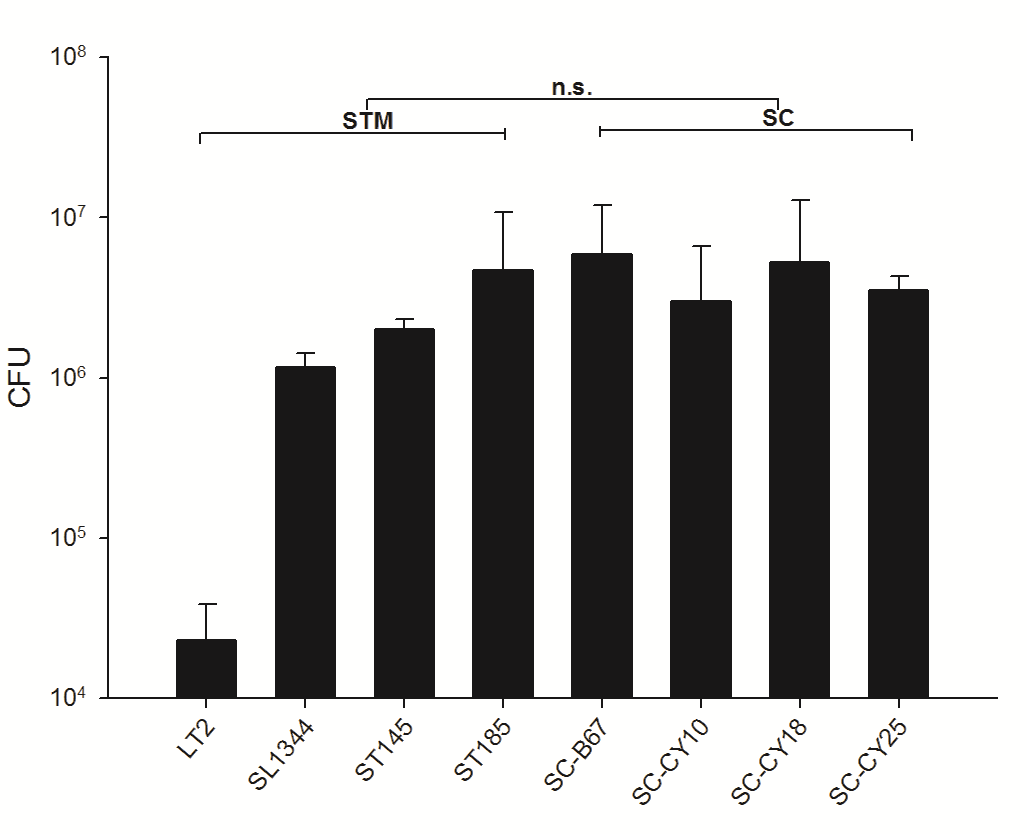


**Supplemental Figure 4.** Epithelial cell invasion assay. HeLa (A) and MDCK (B) cells were infected by *S.* Typhimurium and *S.* Choleraesuis laboratory strains and clinical isolates. After 1.5 hours post-infection, cells were lysed and plated on LB agar. n.s., no significance.

A.


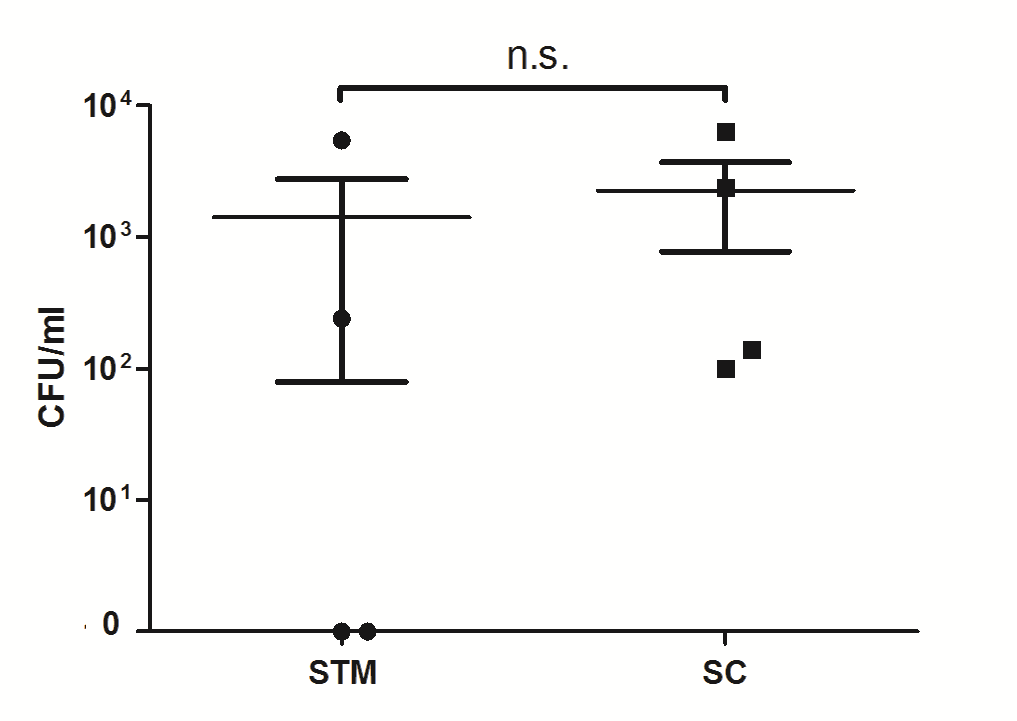


B.


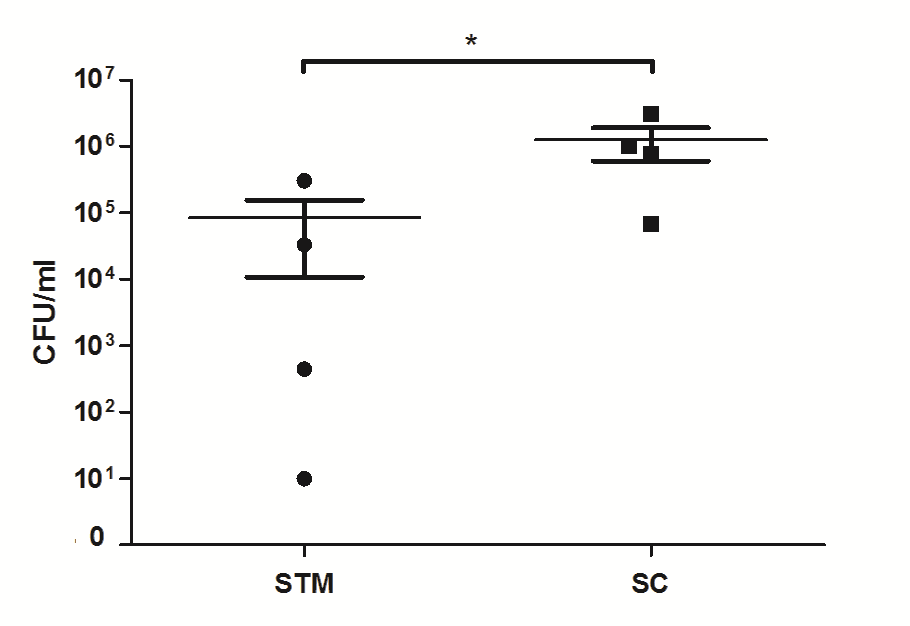


C.


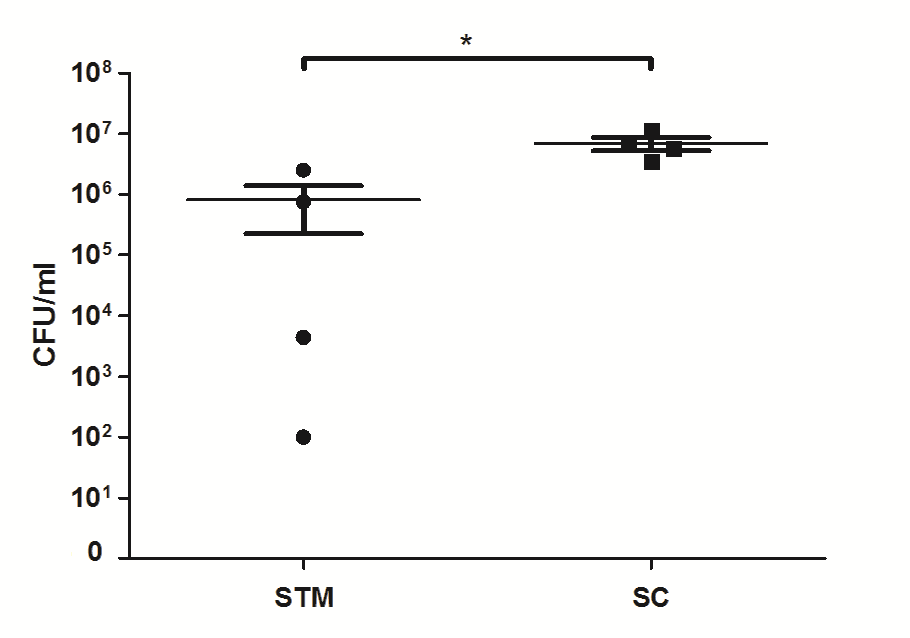


**Supplemental Figure 5.** Cell monolayer penetration assay of *S.* Typhimurium (STM) and *S.* Choleraesuis (SC) laboratory strains and clinical isolates. At 1 hour (A), 3 hours (B), and 6 hours (C) post-infection, the number of *Salmonella* were calculated through plating on the LB agar. n.s., no significance. *, p<0.05

A.


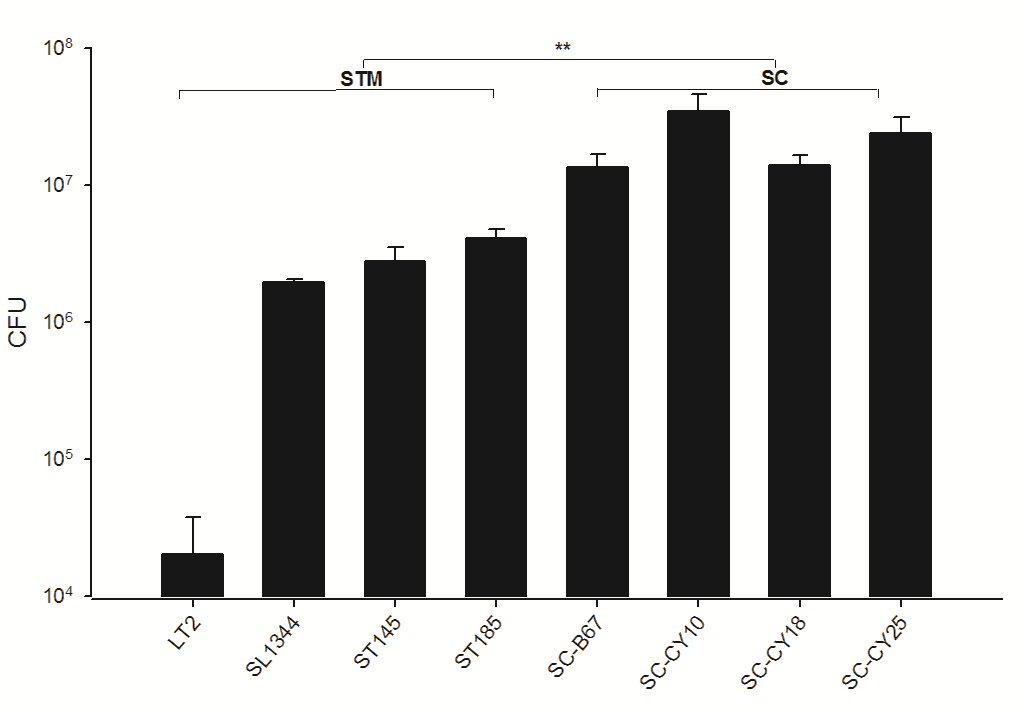


B.


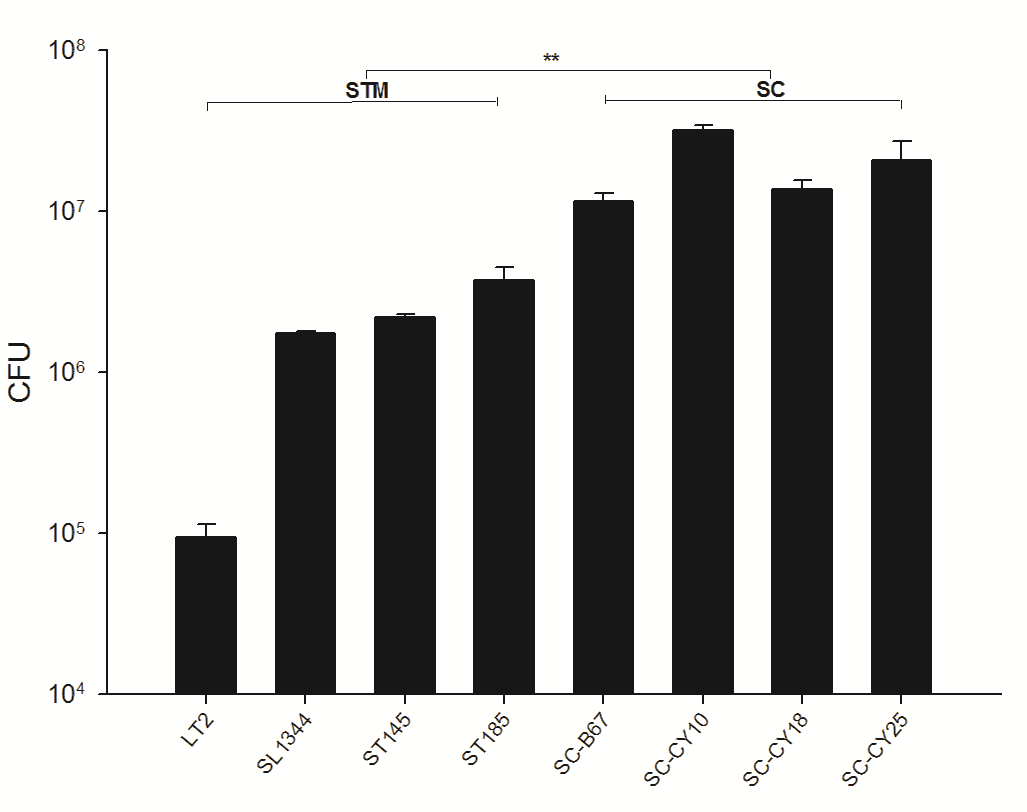


**Supplemental Figure 6.** Intra-macrophage survival assay. THP-1 cells were infected by *S.* Choleraesuis and *S.* Typhimurium laboratory strains and clinical isolates. At 4 hours (A) and 6 hours (B) post-infection, cells were lysed and plated on LB agar and counted. The experiment was repeated 3 times. **, p＜0.01.


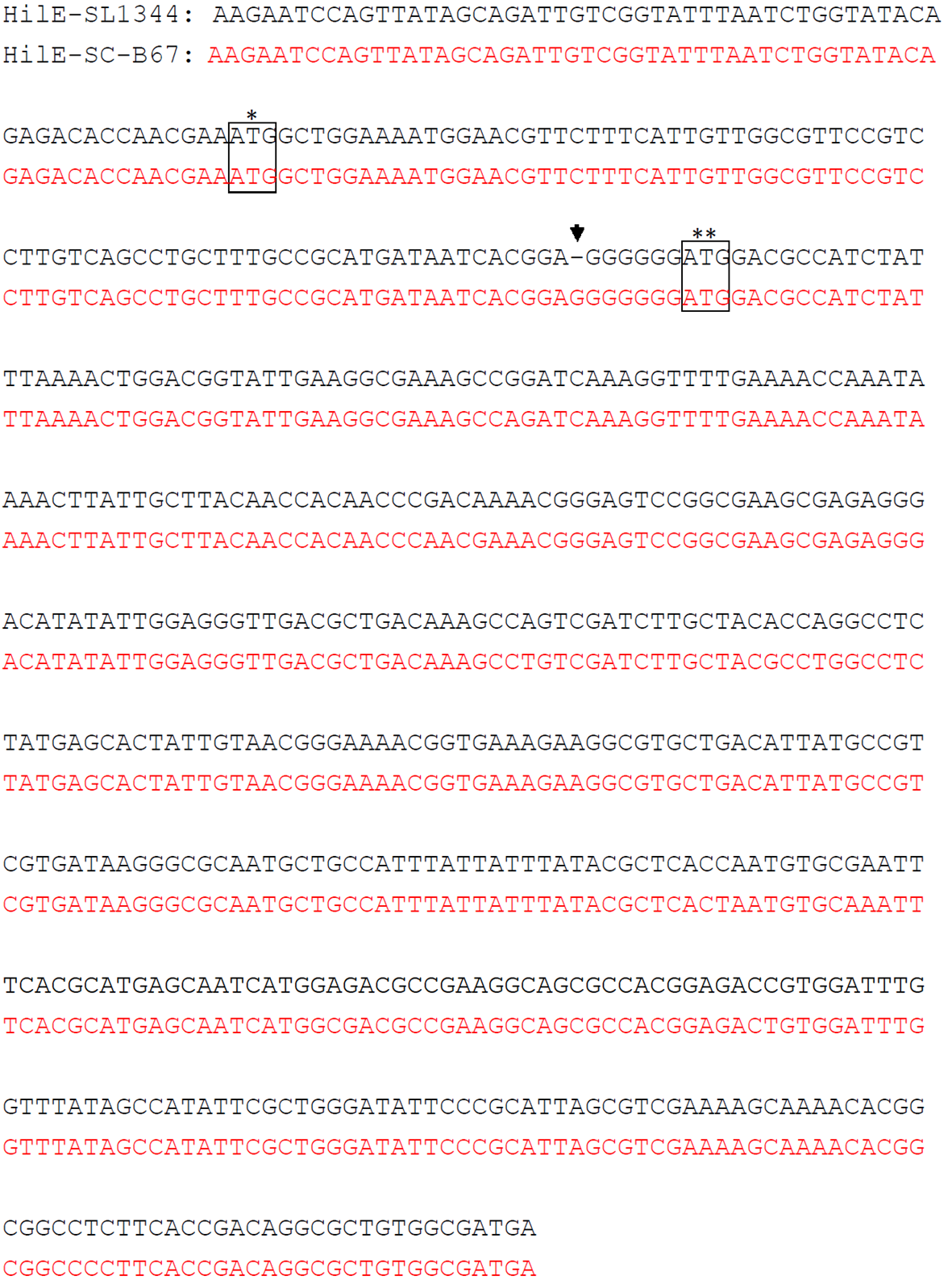


**Supplemental Figure 7.** *hilE* sequencec of *S.* Typhimurium and *S.* Choleraesuis. * and ** indicate the translation start codon of *hilE* in *S.* Choleraesuis and *S.* Typhimurium, respectively. Arrow head indicates the difference between two putative start codons in the two serovars.


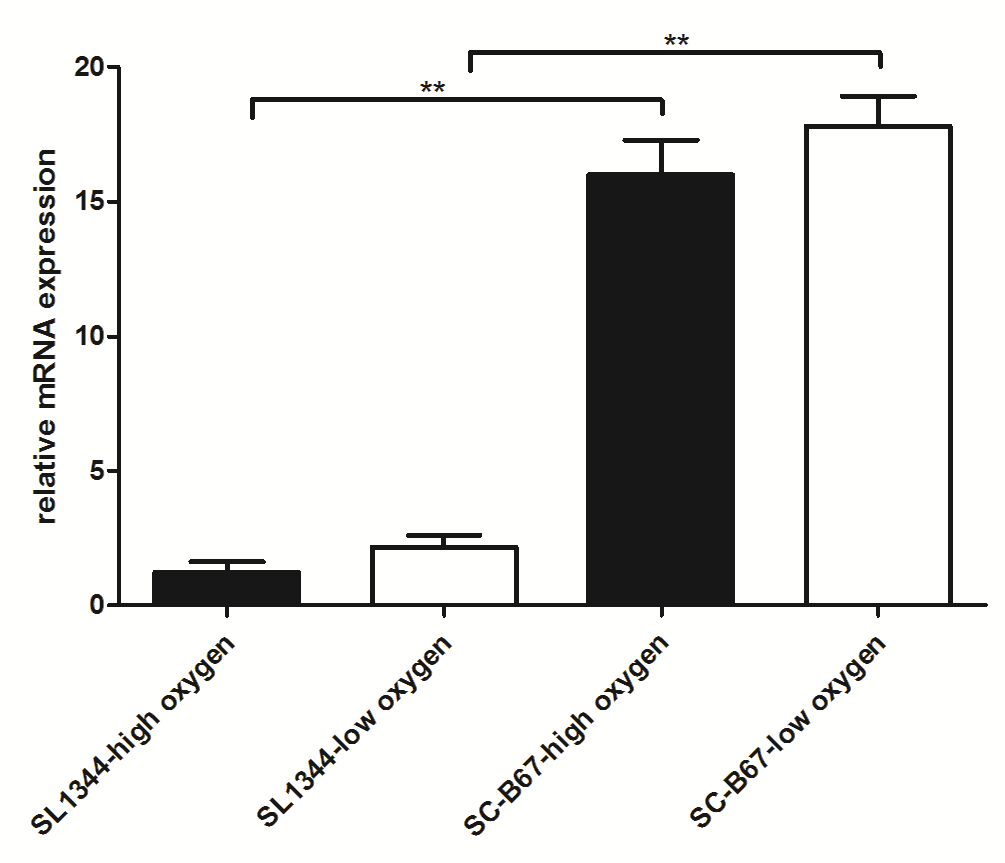


**Supplemental Figure 8.** Expression level of *hilD* in *S*. Typhimurium SL1344 and *S*. Choleraesuis SC-B67 in cell culture medium under high and low oxygen conditions. Both strains were incubated in DMEM with (high oxygen) or without (low oxygen) shaking before detection. Expression level of each gene was normalized with 16S rDNA expression level. **, p<0.01

**Supplemental References**

1. McClelland, M. *et al.* Complete genome sequence of *Salmonella* *enterica* serovar Typhimurium LT2. *Nature* **413**, 852-856 (2001).
2. Hoiseth, S. K. & Stocker, B. A. Aromatic-dependent *Salmonella typhimurium* are non-virulent and effective as live vaccines. *Nature* **291**, 238-239 (1981).
3. Chiu, C. H. *et al.* 2005. The genome sequence of *Salmonella* *enterica* serovar Choleraesuis, a highly invasive and resistant zoonotic pathogen. *Nucleic Acids Res.* **33**, 1690-1698 (2005).
4. Hirakata, Y. *et al.* Pentration of clinical isolates of *Pseudomonas aeruginosa* through MDCK epithelial cell monolayers. *J. Infect. Dis.* **181**, 765-769 (2000).
5. Watson, N. A new revision of the sequence of pBR322. *Gene* **70**, 399-403 (1988).
6. Datsenko, K. A. & Wanner, B. L. One-step inactivation of chromosomal genes in *Escherichia coli* K-12 using PCR products. *Proc. Natl. Acad. Sci. U.S.A.* **97**, 6640-6645 (2000).
